# Development of DNA Aptamers Against *Leishmania infantum* GP63 Protein for Therapeutic and Diagnostic Applications

**DOI:** 10.3390/pharmaceutics18030304

**Published:** 2026-02-28

**Authors:** Lucía Román-Álamo, Daniela Currea-Ayala, Gabriel S. Oliveira, Antonino Nicolò Fallica, Timen Mooren, Yunuen Avalos-Padilla, Xavier Fernàndez-Busquets

**Affiliations:** 1 Barcelona Institute for Global Health (ISGlobal), Hospital Clínic-Universitat de Barcelona, Parc Científic de Barcelona, Baldiri Reixac 10-12, 08028 Barcelona, Spain; dcurrea@ibecbarcelona.eu (D.C.-A.); afallica@ibecbarcelona.eu (A.N.F.); yavalos@ibecbarcelona.eu (Y.A.-P.); 2 Nanomalaria Group, Institute for Bioengineering of Catalonia (IBEC), The Barcelona Institute of Science and Technology, Baldiri Reixac 10-12, 08028 Barcelona, Spain; 3 Doctoral School of Biotechnology, Faculty of Pharmacy and Food Sciences, University of Barcelona, 08028 Barcelona, Spain; 4 Instituto de Investigação e Inovação em Saúde (i3S), Universidade do Porto, 4200-135 Porto, Portugal; jorge.oliveira@i3s.up.pt; 5 Instituto de Ciências Biomédicas Abel Salazar (ICBAS/UP), Universidade do Porto, 4200-135 Porto, Portugal; 6 Biomedical MRI (BMRI), KU Leuven, Herestraat 49, 3000 Leuven, Belgium; timen.mooren@kuleuven.be; 7 Nanoscience and Nanotechnology Institute (IN2UB), University of Barcelona, Martí i Franquès 1, 08028 Barcelona, Spain

**Keywords:** *Leishmania infantum*, leishmaniasis, GP63, DNA aptamers

## Abstract

**Background/Objectives:** Leishmaniasis is a disease affecting millions of people caused by parasites of the genus *Leishmania*. The GP63 protein of *Leishmania infantum* (*Li*GP63) is one of its major surface antigens and a main virulence factor, playing a role in the adhesion of extracellular promastigote stages to macrophages and in the survival of intracellular amastigotes. **Methods:** Here, DNA aptamers have been developed against *Li*GP63 through the systematic evolution of ligands by exponential enrichment. **Results:** Twenty individual aptamer sequences were characterized using confocal fluorescence microscopy and flow cytometry analysis, and 14 of them had targeting to more than 70% of *L. infantum* promastigotes with different subcellular localization patterns. Subsequent dot blot analyses narrowed down the selection to five candidates for further characterization through an aptamer-linked immobilized sorbent assay where it was possible to detect endogenous *Li*GP63 in *L. infantum* promastigote lysates. The five selected aptamers recognized the recombinant *Li*GP63 protein with binding affinities ranging from 0.3 to 2.1 µM. Promastigotes preincubated with *Li*GP63Apt-4, -27 and -28 exhibited a significantly reduced adhesion to and infection of RAW 264.7 macrophages. Moreover, when *Li*GP63Apt-4 and -28 were conjugated to liposomes, these two aptamers significantly enhanced the targeting to *L. infantum* promastigotes compared to plain liposomes. **Conclusions:** Given their improved stability and cost-effectiveness over antibodies, the aptamers evolved here represent promising candidates for new therapeutic and diagnostic approaches and for future nanoparticle-based drug delivery strategies in leishmaniasis.

## 1. Introduction

Over 20 species of *Leishmania* can infect humans, leading to various clinical manifestations that range from self-healing skin lesions in cutaneous leishmaniasis (CL) to potentially fatal visceral leishmaniasis (VL), with disease severity depending on the specific parasite species and the host immune response. Despite over 12 million people being infected worldwide, with an annual incidence of 50,000 to 90,000 and 600,000 to 1 million new cases of VL and CL, respectively, leishmaniasis is still considered a neglected tropical disease [1]. The *Leishmania* life cycle involves two main stages: promastigotes and amastigotes. The flagellated and mobile promastigote resides within the midgut of certain sandfly species and is transmitted to the vertebrate host through a bite. Inside macrophages and other mononuclear phagocytic cells, the parasite transforms into the non-motile amastigote.

The usual diagnosis technique for leishmaniasis is the detection of amastigotes through microscopic examination. For CL this is done in ulcer biopsies, aspirates from the active lesion border, and dermal scrapings, whereas for VL invasive splenic aspirates are required [2]. *Leishmania* antigen detection in serological tests (ELISA, immunofluorescence, Western blot or agglutination analysis) cannot reliably differentiate active from quiescent infection. These tests have a low sensitivity and encounter difficulties for their application in most endemic areas, and the humoral response in CL is poor [3]. Antigen detection tests applied to non-invasively obtained fluids are easier to use and cost-efficient. For instance, an agglutination test was able to identify a urinary *Leishmania* carbohydrate antigen [4], and the ELISA detection of antibodies against the rK28 antigen in the urine of a host for VL diagnosis had a sensitivity similar to the same assay done in serum [5]. The leishmanin skin test is based on a T cell inflammatory reaction when the leishmanin antigen is injected intradermally in the forearm of individuals. It has been widely used in epidemiological studies to detect exposure and immunity to *Leishmania*, and as a complementary diagnostic tool for CL in endemic regions of South America. However, it is no longer used nowadays due to the unavailability of leishmanin obtained under good-practice manufacturing conditions [6]. Rapid diagnostic tests (RDTs) for leishmaniasis offer a varying sensitivity and mainly complement clinical identification, since they do not distinguish between active and past infection [3]. An FDA-approved RDT for CL (CL Detect^TM^ Rapid Test) is based on the detection of peroxidoxin, an antigen expressed in both promastigotes and amastigotes, although one study reported that its sensitivity for detecting CL caused by *Leishmania donovani* was only 36% that of PCR [7]. Regarding the detection of VL, the most widely used RDT is the rK39 test, which detects host antibodies against rK39 antigen, a recombinant fragment of a *Leishmania* kinesin-related protein. However, its sensitivity varies geographically, being lower in East African populations (approximately 85.3%) compared with about 97% in India [8]. Molecular techniques like PCR are sensitive, but they are not routinely used in the field. To overcome this limitation, a reverse transcriptase loop-mediated isothermal amplification assay has been developed as a point-of-care diagnostic tool able to detect all *Leishmania* species in one test using basic equipment [9].

**Figure 1:**
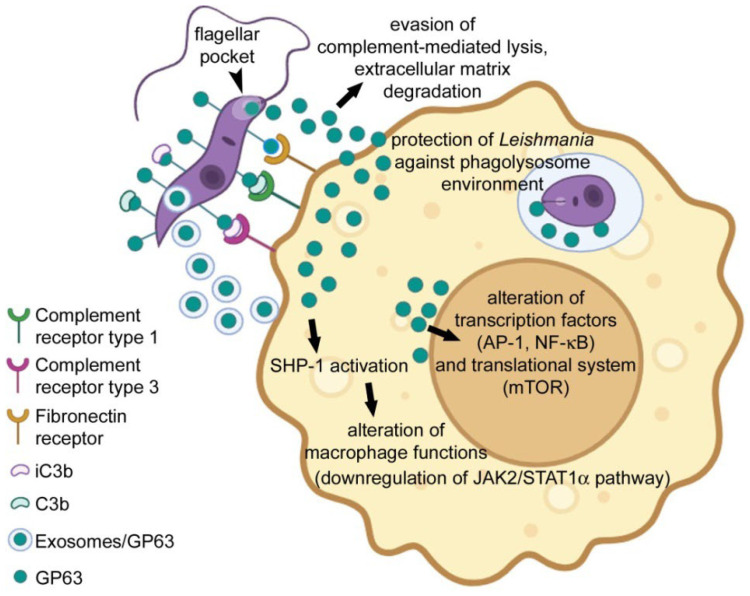
Schematic diagram of GP63 functions in *Leishmania* [16,30]. GP63 facilitates parasite migration through the extracellular matrix by degrading its components and protects *Leishmania* from complement-mediated lysis by cleaving complement component 3b (C3b) into its proteolytically inactive form (iC3b), which can bind to complement receptor types 1 and 3. GP63 binds macrophage fibronectin receptors, promoting adhesion of promastigotes to host cells. GP63 is secreted by promastigotes either via the classical secretory pathway through the flagellar pocket or via exosomes. It is believed that GP63 enters the macrophage cytoplasm through lipid rafts, where it cleaves and activates tyrosine phosphatases, such as SHP-1. This activation downregulates signaling pathways, including JAK2/STAT1α, which leads to an altered macrophage function. GP63 also modulates transcriptional regulation by inhibiting the mTOR pathway and targeting transcription factors such as AP-1 or NF-κB in the perinuclear region. In the amastigote stage, GP63 is thought to protect the parasite from the harsh phagolysosomal environment. Together, these functions promote *Leishmania* survival within host cells [16,30].

The zinc-metalloprotease GP63, or leishmanolysin, is the most abundant protein on the surface of *Leishmania* promastigotes [10] and one of its main virulence factors [11]. GP63 associates with the fibronectin receptors present on the surface of the host macrophage and also cleaves complement component 3b (C3b), which binds to complement receptors. These interactions facilitate the adhesion of the parasites to the macrophages and allow for rapid and efficient internalization [12]. Among the multiple functions of GP63 (Figure 1) are its essential role in amastigote survival following internalization [13]. After phagocytosis, *Leishmania* promastigotes inside the phagosome start modifying it into a parasitophorous vacuole through the participation of pathogenicity factors. One of these is the cysteine proteinase B, whose elimination in *Leishmania mexicana* was reported to induce a T helper 1 response and limited lesion extension in mice [14]. *L. mexicana* virulence was shown to be regulated by GP63, since its exogenous expression restored pathogenicity [15]. In all the *Leishmania* species studied, GP63 has been detected in the amastigote stage [16]. The survival and replication of the parasite in macrophages is facilitated by an inhibition of the release of signaling molecules like TNF, IL-12, IFN-γR and NO [17,18,19,20], which are part of the host response against infection. GP63 triggers a series of signaling modifications that are responsible for this inhibition, such as the activation of protein tyrosine phosphatases in the IRAK-1, MAPK and JAK pathways [18,21,22,23], downregulation of host cell protein synthesis by disrupting mTORC1-dependent signaling [24], inactivation of the transcription factors AP-1 and NF-κB [25,26], or targeting of the nuclear envelope through nucleoporin degradation [27]. In contrast, GP63 cleaves the SNARE protein synaptotagmin XI, a negative regulator of the pro-inflammatory cytokines TNF and IL-6 [28]. Because these cytokines attract to the parasite inoculation site inflammatory monocytes and neutrophils, GP63 action indirectly contributes to infection establishment and spread [29].

From inside the macrophage, *Leishmania* modulates the host adaptive immunity. In addition to its suppressive effects in macrophages, GP63 also targets proteins in other cell types [21,31,32,33], e.g., by altering the expression of surface receptors in NK cells, thus blocking their proliferation [31], and by cleaving the surface receptor CD4 [32], required for T lymphocyte activation. Thus, CD4 cleavage by GP63 will contribute to inhibiting the immune response to antigen-presenting cells. In agreement with this, the GP63 cleavage of VAMP8 has been shown in *L. donovani* and *Leishmania major* to inhibit, in the context of MHC class I, the cross-presentation of pathogen antigens to CD8+ T lymphocytes, resulting in a reduction in their activation [33]. The above evidence places GP63 as a preferred target for future leishmaniasis control efforts.

Single-stranded oligonucleotide DNA aptamers fold into three-dimensional structures capable of binding target molecules or cells with a high affinity and specificity [34,35,36]. Aptamers offer several advantages over antibodies, including a smaller size for enhanced tissue penetration, better stability at room temperature, non-immunogenicity, and animal-free production, which makes them more cost-effective, scalable, and ethically superior. In addition, aptamers can be easily modified to give them a higher resistance to nucleases, extended circulation times, or the potential for incorporating a range of chemical functionalizations for diagnostic [37] and therapeutic uses [38]. In the context of *Leishmania* infection, these properties are important to ensure stability, particularly for therapeutic applications where aptamers would be exposed to biological fluids and macrophage-rich environments. Although diagnostic applications allow a higher experimental flexibility, nuclease activity will remain a relevant factor to take into account. Several aptamers targeting *Leishmania* proteins have been reported for diagnostic applications. Aptamers binding the *Leishmania infantum* kinetoplastid membrane protein 11 (*Li*KMP-11) [39], involved in parasite mobility, were incorporated into an aptasensor able to detect 2.27 µM *Li*KMP-11 [40]. The *L. infantum* histones H2A (*Li*H2A) and H3 (*Li*H3) were used to develop specific aptamers against them, which showed a *K_D_* in the nM range [41], although the limits of detection (7500 parasites for *Li*H2A [42] and 6000 for *Li*H3 [43]) were too high for diagnostic needs. Aptamers selected against *L. major* promastigotes were employed to assemble a fluorescent enzyme-linked aptamer-magnetic bead sandwich assay, which offered a rapid and sensitive detection of promastigote protein extracts in sandfly homogenates [44]. Aptamers developed against the *L. infantum* poly (A)-binding protein, which plays a crucial role in translation, disrupted its interaction with poly(A) tails, representing one of the few examples of the therapeutic potential of aptamers for parasitic diseases [45].

Despite the importance of GP63 in leishmaniasis pathophysiology, no aptamers targeting this protein have been described so far. In this work, DNA aptamers against *L. infantum* GP63 (*Li*GP63) have been generated and evaluated for their potential application in diagnostic and prophylactic approaches. Because GP63 is highly conserved among *Leishmania* species, targeting *Li*GP63 provides a biologically relevant basis for developing pan-antileishmanial strategies.

## 2. Materials and Methods

### 2.1. Reagents

Except where otherwise indicated, reagents were purchased from Sigma-Aldrich Corporation (St. Louis, MO, USA), and reactions were performed at room temperature (22 to 24 °C).

### 2.2. Protein Expression and Purification

The region encoding the mature form of *L. infantum* GP63-2 (*Li*GP63m, LINF_100010200), spanning residues Val97 to Asn574 of the full-length protein, was PCR-amplified from genomic DNA extracted from a *L. infantum* culture with the DNeasy^®^ Blood & Tissue kit (Qiagen, Venlo, The Netherlands), according to the manufacturer′s protocol. The selected primers (forward: 5′GAAGGTCGTGGGATCGTCGTGCGCGCCGCGAACTGG3′, reverse: 5′GGCCGCTCGAGTCGAGTTGCCGCCGTCCTTGGC3′) allowed the seamless cloning of the gene into the *Bam*HI and *Sal*I sites of the pGEX-5X-1 plasmid using the InFusion cloning system (Clontech, Kusatsu, Japan), following the manufacturer’s instructions. For glutathione-S-transferase (GST) purification, the empty pGEX-5X-1 plasmid was used. After confirming through Sanger sequencing the insertion of the *Li*GP63m gene (1433 bp), *Escherichia coli* C41 competent cells were transformed with either pGEX-5X-1 or pGEX-5X-1/*Li*GP63m constructs. The protein expression of GST and GST-*Li*GP63m was induced by adding 0.1 mM isopropyl β-D-thiogalactoside (IPTG) to a transformed C41 *E. coli* culture during the logarithmic growth phase. Following this, the culture was incubated for 16 h at 20 °C in Terrific Broth medium (TB: 24 g/L yeast extract, 20 g/L tryptone, 4 mL/L glycerol, 10% potassium phosphate (0.17 M monobasic, 0.72 M dibasic), 1% glucose) supplemented with 0.1 mg/mL ampicillin. To prepare the cell lysate, bacteria were subjected to sonication in lysis buffer 1 (300 mM NaCl, 1 mM ethylenediaminetetraacetic acid (EDTA), 1 mM PEFABLOC SC (Roche, Basel, Switzerland), 2 mg/mL lysozyme, 50 mM tris-HCl, pH 8.0) and centrifuged at 16,000× *g* for 90 min at 4 °C to separate insoluble from soluble fractions. In the case of GST-*Li*GP63m, as it remained in the insoluble fraction, the protein was extracted from inclusion bodies using two rounds of solubilization buffer. First, the cell pellet was suspended in buffer 1 (2 M urea, 500 mM NaCl, 2% Triton X-100, 1 mM EDTA, 20 mM tris-HCl, pH 8.0), sonicated (8 min, 30 s on, 30 s off, amplification 80%, 4 °C) with a Sonics Materials VCX-130PB Ultrasonic Processor (Thermo Fisher Scientific, Waltham, MA, USA) and centrifuged as above. The new resulting pellet was then resuspended in buffer 2 (4 M urea, 500 mM NaCl, 2% Triton X-100, 1 mM EDTA, 20 mM tris-HCl, pH 8.0), sonicated and centrifuged again with the same settings as before. The solubilized protein fraction was dialyzed using Pur-A-Lyzer^TM^ Mega Dialysis Kit (Sigma-Aldrich, St. Louis, MO, USA) against decreasing concentrations of urea to allow refolding of the protein. GST and refolded GST-*Li*GP63m were then purified through affinity chromatography using Glutathione Sepharose^®^ 4B beads (Cytiva, Marlborough, MA, USA), according to the manufacturer’s protocol. Briefly, 500 µL of a 50% washed bead slurry was incubated overnight at 4 °C under gentle stirring with each of the proteins. After this time, beads carrying GST and GST-*Li*GP63m were washed and aliquoted (at, respectively, 2 × 10^5^ and 1 × 10^5^ beads/vial, which bound ca. 21 and 15 µg of protein), and frozen at −20 °C until their use. To confirm the presence and integrity of GST and GST-*Li*GP63m, 10 µL of protein-carrying beads was incubated for 5 min at 95 °C diluted in Laemmli solution (0.14 M sodium dodecyl sulfate (SDS), 20% glycerol, 10% 2-mercaptoethanol, 3 mM bromophenol blue, 0.125 M tris-HCl, pH 6.8) and resolved in a 10% SDS-polyacrylamide gel electrophoresis (SDS-PAGE; Mini Protean II System, Bio-Rad Laboratories, Inc., Hercules, CA, USA) stained with Coomassie blue.

To obtain larger protein amounts for binding affinity characterization, and to have *Li*GP63m with a tag different from GST in order to confirm aptamer specificity, the gene encoding *Li*GP63m was cloned into the pCold^TM^ TF DNA plasmid (Takara Bio, Kusatsu, Japan), using the InFusion cloning system, to obtain a 6× histidine-tagged protein (forward and reverse primers used were 5′TACCCTCGAGGGATCCGTCGTGCGCGCCGCGAACTGG3′ and 5′TAGACTGCAGGTCGACGTTGCCGCCGTCCTTGGC3′, respectively). After confirming the insertion of the gene through Sanger sequencing, *E. coli* C43 competent cells were transformed with the pColdTF-*Li*GP63m construct, and the expression of the protein (His-TF-*Li*GP63m) was induced through the addition of 1 mM IPTG to bacteria cultured in TB medium supplemented with 0.1 mg/mL ampicillin, followed by incubation for 24 h at 15 °C. Cells were lysed through sonication in lysis buffer 2 (500 mM NaCl, 20 mM imidazole, 1 mM PEFABLOC SC, 2 mg/mL lysozyme, 20 µg/mL DNAse I, 1 mM MgCl_2_, 20 mM sodium phosphate, pH 7.4) and centrifuged (16,000× *g*, 1 h, 4 °C) to separate the insoluble from the soluble fraction. The His-tagged protein was subsequently purified from the soluble fraction using a HisTrap HP His tag protein purification column (Cytiva) connected to an ÄKTA pure^TM^ chromatography system (Cytiva). The purified protein was eluted with increasing concentrations of imidazole (up to 500 mM), and positive fractions were buffer exchanged with phosphate-buffered saline, pH 7.4 (PBS), using PD-10 desalting columns (GE Healthcare, Chicago, IL, USA), and concentrated with an Amicon^®^ Ultra-4, NMWL 10 kDa (Merck, Darmstadt, Germany). Protein aliquots were quantified as described above and maintained at −20 °C until use. The successful expression and purification of the His-TF-*Li*GP63m was corroborated in an SDS-PAGE analysis performed as detailed above.

### 2.3. Generation of DNA Aptamers Against LiGP63m

DNA aptamers against recombinant *Li*GP63m were obtained using the systematic evolution of ligands through the exponential enrichment (SELEX) method [46,47], starting from an ssDNA random library pool (DNA Technology A/S, Denmark). Each ssDNA sequence in the pool was 76 bases long (ATACCAGCTTATTCAATTNNNNNNNNNNNNNNNNNNNNNNNNNNNNNNNNNNNNNNNNAGATAGTAAGTGCAATCT), of which the central 40 were randomized, and the two 18-base ends were complementary to the forward and reverse primers used for PCR amplification, which were tagged at their 5’ ends with 6-FAM and dual biotin, respectively. In the first cycle, 10 nmol of DNA library in 1 mL of binding buffer (PBS containing 1 mg/mL of bovine serum albumin (BSA)) was mixed and heated to 95 °C for 5 min and then cooled in ice for 10 min to allow ssDNA folding. The oligonucleotides were then incubated with 2 × 10^5^ GST-Sepharose beads for 1 h under stirring at room temperature (counter selection). Non-binding sequences were then incubated with 2 × 10^5^ GST-*Li*GP63m-beads in the same conditions. In this case, the non-binding ssDNAs present in the supernatant after a centrifugation (500× *g*, 5 min) were removed. The resulting bead pellet was taken up in 300 µL of binding buffer and incubated at 95 °C for 10 min to release the bound oligonucleotides, which were separated from the beads through centrifugation as above. To regenerate a sufficient amount of ssDNA for the next SELEX cycle, PCR amplification was performed after each selection using the 2× PCR Master Mix (Thermo Fisher Scientific). The amplified DNA was precipitated through the addition of 0.1 vol of 3 M sodium acetate and 2.5 vol of cold absolute ethanol. After thoroughly mixing, the tubes were stored at −20 °C for at least 30 min and centrifuged (20,100× *g*, 40 min, 4 °C). The resulting pellet was washed with 70% ethanol, centrifuged as before, and the solvent was evaporated with a SpeedVac concentrator (SPD 1010, Thermo Scientific Savant) for 35 min. The dsDNA was taken up in PBS and quantified in a Nanodrop 2000 (Thermo Fisher Scientific), and the forward single strand was purified in Micro Bio-Spin chromatography columns (Bio-Rad Laboratories, Inc.) loaded with High Capacity NeutrAvidin^TM^ Agarose (Thermo Fisher Scientific), which bound the dual-biotin tag in the reverse chain. Following the manufacturer’s instructions, fluorescein-ssDNA was eluted with 500 µL of 0.1 M NaOH. To neutralize the base, 50 µL of 1 M HCl was added to each elution. The resulting ssDNA was precipitated as above, taken up in 200 µL of binding buffer, and incubated again with 2 × 10^5^ GST-*Li*GP63m-beads to start the next round of selection. The amount of GST-*Li*GP63m-beads used from cycle 1 to 3 was 2 × 10^5^, whereas from cycle 4 onwards, a concentration of 1 × 10^5^ beads was used to increase the stringency of the selection. Seven SELEX cycles were performed, and a counter-selection with 1 × 10^5^ protein-free GST-Sepharose beads was done every three cycles to remove unspecific binding sequences.

### 2.4. Selection of Individual Aptamer Sequences

The ssDNA in the oligonucleotide pool obtained after 7 SELEX cycles was PCR-amplified with unlabelled forward and reverse primers using a high-fidelity *Pfu* DNA polymerase (BioTools, South San Francisco, CA, USA). The resulting dsDNA was cloned into the pJET1.2/blunt plasmid according to the manufacturer’s instructions, and transformed into competent *E. coli* DH5α cells (Thermo Fisher Scientific), which were seeded in Luria Broth (LB) containing 100 µg/mL ampicillin agar plates and incubated overnight at 37 °C. Thirty colonies were selected for plasmid extraction and purification using the GeneJET mini-prep kit (Thermo Fisher Scientific), following the manufacturer’s protocol. The inserts were sequenced using GENEWIZ (Azenta Life Sciences, Burlington, MA, USA), and the resulting aptamer sequences were analyzed with the QGRS Mapper 2 software (https://bioinformatics.ramapo.edu/QGRS/index.php, accessed on 23 February 2024) to predict the presence of G-quadruplexes.

### 2.5. G-Quadruplex Detection by Thioflavin-T

The experimental procedure reported by Li et al. [48] was followed with minor modifications. Aptamer samples were diluted to a final concentration of 1 µM in a 10 mM tris-HCl buffer (pH 7.4) containing 100 mM KCl. Aptamer folding was promoted by heating the samples at 95 °C for 5 min, followed by slow cooling to room temperature over 2 h. The resulting mixtures were gently resuspended by pipetting and aliquoted in triplicate into a 96-well black plate (Greiner Bio-One, Madrid, Spain) to a final volume of 100 µL/well to which a freshly prepared solution of thioflavin T (ThT) was added to a final concentration of 1 µM. The plate was gently shaken for 15 min at room temperature in the dark, and ThT fluorescence was measured using a Tecan Infinite^®^ M Plex microplate reader, with an excitation wavelength of 450 nm and emission collected between 480 and 800 nm. The obtained fluorescence values were corrected subtracting the signal of a ThT blank prepared in the same buffer used for the aptamer-containing samples. Data are reported as the mean ± SEM of three independent experiments.

### 2.6. Confocal Fluorescence Microscopy and Flow Cytometry Analysis

*L. infantum* promastigotes of the MHOM/ES/2016/CATB101 strain were fixed with 4% paraformaldehyde (PFA) for 10 min and subsequently washed three times with PBS. Then, 2 × 10^7^ promastigotes were incubated overnight in binding buffer at 4 °C with previously folded 1 µM individual aptamers or 0.2 µM aptamer pools from cycles 2 and 7. To allow a correct pre-folding, aptamers had been incubated in binding buffer as follows: 10 min at 90 °C, 15 min at 5 °C, 8 min at 37 °C, and finally left at 4 °C until use. Promastigotes were then washed with PBS, and nuclei were counterstained for 30 min with 4 µg/µL Hoechst 33342 and rinsed with PBS. For confocal fluorescence analysis, samples were placed in 8-well chamber slides (ibidi GmbH, Gräfelfing, Germany) and observed in a Zeiss LSM 800 confocal microscope (Zeiss, Oberkochen, Germany) with a 100×/1.4 oil DIC M27 objective. Hoechst 33342 and fluorescein were excited with 405 nm and 488 nm diode lasers, and emission was collected in the 390–460 and 500–700 nm ranges, respectively. Manders’ overlap coefficient [49] was calculated by analyzing images with the Coloc 2 plugin in Fiji (version 1.54p) [50]. This coefficient varies from 0 to 1, where 0 corresponds to no overlap between the two signals and 1 represents complete overlap, reflecting the proportion of pixels shared by both channels. For flow cytometry analysis, samples were diluted 1:25 with PBS, and targeting was analyzed in a five-laser LSRFortessa flow cytometer (BD Biosciences, San Jose, CA, USA) in the 20-parameter standard configuration. A total of 50,000 events were recorded, and the promastigote population was selected on a forward-side scattering dot plot. The lasers used to excite Hoechst 33342 and 6-FAM were, respectively, 350 and 488 nm, and the corresponding emissions were collected with 450/50 and 525/50 nm bandpass filters. An unspecific 6-FAM-labeled aptamer (Apt700, [34]) and parasites stained only with Hoechst 33342 were used as negative controls.

### 2.7. Dot Blots and Western Blots

*L. infantum* cultures of the MHOM/ES/2016/CATB101 strain containing 1.5 × 10^7^ promastigotes/mL in the stationary phase were pelleted through centrifugation (600× *g*, 3 min) and washed three times in PBS supplemented with 1× cOmplete^TM^ protease inhibitor cocktail (Roche). Promastigote suspensions were diluted 1:100 in 4% PFA and counted in a Neubauer chamber. To obtain a whole parasite lysate, promastigotes were frozen at −80 °C for 24 h and then thawed. In parallel, 200 µL of 1% Triton X-100 in PBS supplemented with 1× cOmplete^TM^ was added to a different promastigote pellet and incubated for 30 min at 4 °C. Then, the samples were centrifuged (20,000× *g*, 30 min, 4 °C) and the supernatant was recovered (Triton X-100 extract). The resulting pellet was further washed two times with PBS supplemented with 1× cOmplete^TM^ and suspended in 300 µL of radioimmunoprecipitation assay (RIPA) buffer (150 mM NaCl, 1% Triton X-100, 0.1% SDS, 2 mM EDTA, 5% glycerol, 50 mM tris-HCl, pH 9.4) supplemented with 1× cOmplete^TM^. After 15 min of incubation at 4 °C, the sample was centrifuged (20,000× *g*, 5 min, 4 °C), and the resulting supernatant constituted the RIPA extract. The protein concentration of all the samples was determined with the Pierce BCA Protein Assay Kit (Thermo Fisher Scientific). For dot blots, 1.25 or 2.4 µg of protein sample in 2.5 or 5 µL of PBS was deposited onto an Amersham Hybond ECL nitrocellulose membrane (GE Healthcare), and dots were air-dried. The membrane was then blocked overnight with tris-buffered saline (TBS) containing 0.1% Tween^®^ 20 (TBSt) supplemented with 3% *w*/*v* skim milk, washed with TBSt, and incubated with 600 nM 6-FAM-labeled pre-folded aptamers (in TBSt supplemented with 3% *w*/*v* skim milk) for 1 h at room temperature, with gentle stirring. Control membranes were probed with monoclonal mouse anti-His tag (Ref. MAB050, R&D Systems, Minneapolis, MN, USA; 1:2500 dilution in TBSt), monoclonal mouse anti-tubulin (Ref. T9026, Sigma-Aldrich; 1:1000 dilution in TBSt containing 3% *w*/*v* skim milk), and polyclonal goat anti-GST (Ref. GE27-4577-01, Sigma-Aldrich; 1:10,000 dilution in TBSt containing 3% *w*/*v* skim milk) antibodies for 1 h at 37 °C. After this time, membranes were washed 3× with TBSt, incubated for 1 h at room temperature with anti-mouse or anti-goat horseradish peroxidase-conjugated secondary antibodies (Sigma-Aldrich Refs. 12-349 and A8919, respectively; both at a1:10,000 dilution in TBSt), and developed with the ECL Prime Western Blotting Detection Reagent (GE Healthcare). The fluorescence of 6-FAM-labeled aptamers and the peroxidase chemiluminescent signal were detected with an ImageQuant^TM^ LAS 4000 image reader (GE Healthcare), using the SYBR green fluorescence filter and the chemiluminescent method, respectively. When required, the density of the dots was quantified using ImageJ software (version 1.54p) [50]. Data were normalized by subtracting the signal from Apt700, used as a negative control, and using as reference the intensity obtained with the antibody anti-His tag, which was established as 100% of signal.

For Western blot analysis, 1 and 5 μg of the purified recombinant GST-*Li*GP63m and His-TF-*Li*GP63m proteins, respectively, were resolved in a 10% SDS-PAGE, transferred to a nitrocellulose membrane and blocked with 5% *w*/*v* skim milk dissolved in TBS containing 0.5% Tween^®^ 20. Membranes were probed with either anti-GST or anti-His tag, as for dot blot assays.

### 2.8. Determination of Apparent K_D_ and Apparent B_max_

Amounts of 50 µg/mL of purified His-TF-*Li*GP63m and GST, in PBS volumes of 4 and 1 mL, respectively, were combined with aldehyde/sulfate latex beads, 4 µm, 4% *w*/*v* (Thermo Fisher Scientific) at a concentration of 0.052% (*v*/*v*). Following an overnight incubation at 4 °C with gentle stirring, beads were pelleted (three PBS washes, 3000× *g*, 15 min, 4 °C) and taken up with fresh PBS in the original volumes. Subsequently, 50 µL of His-TF-*Li*GP63m-beads (containing 50 µg/mL protein) was mixed with 50 µL of 6-FAM-labeled pre-folded aptamers at seven different concentrations in the range from 2000 to 62.5 nM and incubated overnight at 4 °C under gentle stirring. After one PBS wash, cells were diluted 1:5 in PBS immediately before analysis in a five-laser LSRFortessa flow cytometer, as described above for fluorescein. Control aldehyde/sulfate latex beads carrying GST were incubated only with the two highest aptamer concentrations, and Apt700 served as an additional negative control. Mean fluorescence intensity was determined using FACSDiva Version 6.1.3 software (BD Biosciences). The apparent maximum number of protein binding sites (*B_max_*), the apparent aptamer concentration that binds to half the receptor sites at equilibrium (*K_D_*), and the ratio between them (binding potential) were obtained by fitting the intensity of specific binding to the aptamer concentration using the equation Y = *B_max_* X/(*K_D_* + X). GraphPad Prism 9 (GraphPad Software, San Diego, CA, USA) was used to generate the saturation curve, with non-linear regression fit for binding analysis, selecting a one-site binding equation.

### 2.9. Binding Affinity Assays

The aptamer-linked immobilized sorbent assay (ALISA, [51]) was used to determine the limit of detection of the aptamers for the recombinant His-TF-*Li*GP63m and for *Li*GP63 in whole parasite lysates. His-TF-*Li*GP63m and GST were diluted at 3.6 µg/100 µL in carbonate–bicarbonate buffer (pH 9.6), and seven 1:2 serial dilutions were deposited for overnight immobilization at 4 °C in 96-well immunoassay plates with transparent flat bottoms (Thermo Fisher Scientific). The volume added per well was 50 µL, and therefore the highest amount of protein in the assay was 1.8 µg. Then, samples were washed three times with PBS containing 0.05% Tween^®^ 20 (PBSt) and subsequently blocked for 1 h at 37 °C with 100 µL/well of PBS containing 3% BSA. In parallel, 6-FAM-labeled aptamers (15 µM) were pre-folded as described above, diluted in PBSt to a concentration of 600 nM, and 50 µL/well was added after removing the blocking solution. Following an overnight incubation at 4 °C and two washes with PBSt, the fluorescence emission of 6-FAM was collected at 530 nm using an excitation wavelength of 488 nm (Infinite Nano+ multimode microplate reader; Tecan Trading AG, Männedorf, Switzerland). Binding affinity to whole parasite lysates was done as for His-TF-*Li*GP63m except for the incubation time with the aptamers (1 h at room temperature), the number of PBSt washes (3) before fluorescence reading, and the amount of total protein/well (0.11 µg to 3.5 ng, corresponding to ca. 30,000 and 938 *L. infantum* promastigotes, respectively).

### 2.10. Effect of Aptamers on L. infantum Adhesion to and Infection of Macrophages

RAW 264.7 macrophages were seeded in an 8-well chamber slide (ibidi GmbH) at 10^5^ cells/mL in Dulbecco’s modified Eagle’s medium (DMEM) supplemented with 10% fetal bovine serum (FBS) and 1% penicillin–streptomycin in a final volume of 300 µL per well, and left to adhere overnight. Meanwhile, aptamers *Li*GP63Apt-4 and -28 and Apt700 were folded as previously described, and 1 µM of each was incubated with 10^6^ promastigotes/mL in a final volume of 300 µL of DMEM supplemented with 2% FBS and 1% penicillin–streptomycin. After 1 h of incubation, promastigotes were centrifuged (680× *g*, 3 min, 2×), and the final pellet was taken up in DMEM supplemented with 2% FBS and 1% penicillin–streptomycin and added to the adhered RAW 264.7. After an overnight incubation, cells were washed with PBS five times to remove extracellular parasites. Four µg/mL of Hoechst 33342 and 5 µg/mL of wheat germ agglutinin (WGA)-Alexa Fluor 647 (Thermo Fisher Scientific) were added and incubated for 10 min; then, the cells were washed twice with PBS, followed by fixation with 4% PFA for 15 min and three last washes to remove PFA. Samples were visualized in a Leica TCS SP5 laser scanning confocal fluorescence microscope (Leica Microsystems, Wetzlar, Germany). Hoechst 33342 and WGA-Alexa Fluor 647 were detected through excitation using 405 and 532 nm solid-state lasers, and the respective emissions were collected in the 408–521 and 605–707 nm ranges. Four mosaics were built, each containing 5 × 5 fields for each sample. Parasite adhesion and infection were quantified by counting 100 RAW 264.7 cells per sample. For adhesion studies, the number of macrophages with adhered parasites and total adhered parasites were recorded to calculate the average adhered parasites per adhered macrophage and the adhesion rate. Adhesion rate was defined as the proportion of macrophages with at least one promastigote adhered to their surface relative to the total number of macrophages counted. For infection studies, the number of infected macrophages and total intracellular parasites were recorded to calculate the average parasite load per infected macrophage and the infection rate. Infection rate was defined as the ratio between the number of macrophages carrying at least one intracellular amastigote and the total number of macrophages. Cell counting was carried out with the Fiji software [50]. As controls, each 8-well chamber slide included one well containing uninfected RAW 264.7 cells and one well containing RAW 264.7 cells infected with parasites that had not been pre-incubated with aptamers.

### 2.11. Preparation of Maleimide-Functionalized Liposomes

Liposomes were prepared using the thin lipid film hydration method [52]. Briefly, the lipids (all from Avanti Polar Lipids Inc., Alabaster, AL, USA) 1,2-dioleoyl-*sn*-glycero-3-phosphocholine (DOPC), 1,2-distearoyl-*sn*-glycero-3-phosphoethanolamine-*N*-(maleimide(polyethylene glycol)-2000) (DSPE-PEG-Mal), cholesterol, and carboxyfluorescein-phosphatidylethanolamine (CF-PE) (74.5:5:20:0.5 molar ratio) were mixed in chloroform in a glass vial. The thin film obtained following the evaporation of chloroform under a nitrogen stream was hydrated in the PBS volume necessary to obtain a final lipid concentration of 10 mM, followed by three rounds of constant vortexing for 2 min, and bath sonication at 35 °C for 3 min. Then, liposomes were extruded through 200 nm polycarbonate membranes with a mini extruder device (Avanti Polar Lipids Inc.), and stored at 4 °C until used. The conjugation of aptamers to liposomes was performed using the thiol-maleimide cross-linking reaction between the maleimide group in DSPE-PEG-Mal and the C6-thiol-modified aptamers in their 5’ end (Integrated DNA Technologies, Coralville, IA, USA). Prior to conjugation with liposomes, aptamers were subjected to a chemical reduction where 100 μM aptamer was treated with 10 mM dithiothreitol (DTT) for 1 h at room temperature. Then, the solution was passed through a Zeba^TM^ Spin Desalting Column (Thermo Fisher Scientific) to remove unreacted DTT. The purified aptamers (12.5 µM) were pre-folded as described above and conjugated to maleimide-containing liposomes (1:40 aptamer:DSPE-PEG-Mal ratio) by incubation overnight at 4 °C, followed by ultracentrifugation to remove free aptamers from the liposome suspension. The size, polydispersity index and zeta potential of liposomes (diluted 1:100 in PBS) were measured in a Zetasizer NanoZS90 (Malvern Ltd., Malvern, UK). The amount of DNA in aptamer-functionalized liposomes was calculated using a Nanodrop 2000, following measurements of 260/280 and 260/230 ratios. Data were normalized using the values obtained for plain liposomes.

### 2.12. Targeting of Aptamer-Functionalized Liposomes to L. infantum Promastigotes

For targeting assays, 2 × 10^7^ promastigotes/mL were washed three times with PBS through centrifugation (680× *g*, 3 min) and subsequently fixed for 15 min with 200 µL of 4% PFA. Promastigotes were then washed as above to remove PFA, and the final pellet containing promastigotes was suspended in PBS. A volume of 10 µL of fluorescent liposomes (plain, LP-*Li*GP63Apt-4, and LP-*Li*GP63Apt-28) was added to 2 × 10^6^ fixed promastigotes (100 µL final volume) and incubated for 3 h. During the last 10 min of incubation, Hoechst 33342 was added at a final concentration of 4 µg/µL. Promastigotes were then washed three times, the pellet was taken up in 100 µL of PBS, and a 1:10 dilution in PBS was done and analyzed in a five-laser LSRFortessa flow cytometer with a 20-parameter standard configuration. A total of 10,000 events per sample were recorded, and the promastigote population was selected based on the forward- and side-scattering intensities, having as reference parasites stained only with Hoechst 33342. The excitation of Hoechst 33342 and carboxyfluorescein was done with 350 and 488 nm lasers, respectively, and the corresponding fluorescence emissions were collected with 450/50 and 525/50 nm bandpass filters.

### 2.13. Promastigote Growth Inhibition Assay

Promastigotes were grown at 26 °C in complete Schneider’s medium (supplemented with 10% FBS, 1% penicillin–streptomycin and 1% sterile human urine) in 96-well microtiter plates (Nunclon Delta surface; Thermo Fisher Scientific). Serial 1:2 dilutions of the five pre-folded aptamers (*Li*GP63Apt-4, -10, -23, -27 and -28) were done in complete Schneider’s medium (volume per well: 100 µL), starting with a maximum concentration of 5 µM for each individual aptamer and 2.5 µM for amphotericin B. Subsequently, promastigotes from a culture in the logarithmic growth phase were centrifuged for 3 min at 680× *g*, and the pellet containing the parasites was suspended in 1 mL of complete Schneider’s medium. A 1:100 dilution was made with 4% PFA to determine promastigote concentration in a Neubauer counting chamber. Once the concentration was determined, 100 µL of complete Schneider’s medium containing 2 × 10^6^ promastigotes/mL was added to each well. The plate was incubated at 26 °C for 48 h, after which 0.00125% resazurin sodium salt was added. After a further 24 h, resorufin fluorescence was measured (λex/em: 535/590 nm) in a Tecan Infinite 200 PRO equipment (Tecan Trading AG).

### 2.14. Statistical Data Analysis

Unless otherwise indicated, experiments were performed in triplicate, and the results are expressed as mean values ± standard error of the mean (SEM). Differences between samples were analyzed through one-way analysis of variance using GraphPad Prism 8.4 (GraphPad software, La Jolla, CA, USA), considering a significant *p*-value < 0.05. Mean values and SEM were calculated using Microsoft Office Excel version 2306.

## 3. Results

### 3.1. Aptamer Selection Against L. infantum GP63

**Figure 2:**
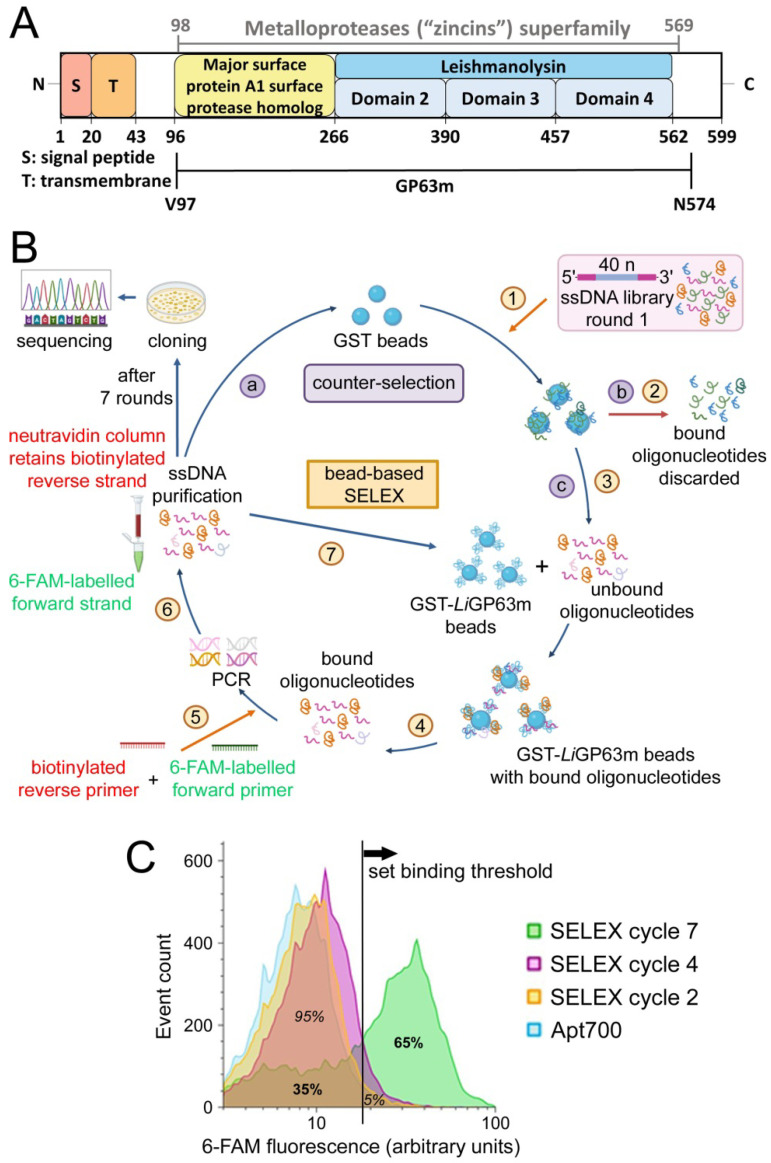
Strategy followed for the selection of aptamers against *L. infantum* GP63. (**A**) Domain scheme of *L. infantum* GP63-2 indicating the *Li*GP63m region used for aptamer selection. (**B**) Diagram of the bead-based SELEX process used to obtain aptamers against GST-*Li*GP63m. The methodology involves iterative cycles of: (1) incubation of GST-beads with the initial ssDNA library, (2) removal of the oligonucleotides bound to GST-beads, (3) incubation of GST-beads–unbound oligonucleotides with GST-*Li*GP63m beads, (4) extraction of the oligonucleotides bound to target beads, (5) amplification of the bound oligonucleotides through PCR with reverse and forward primers labeled on their 5’ ends with biotin and 6-FAM, respectively, (6) purification of the 6-FAM-labeled DNA strand upon retention of the biotinylated strand in a neutravidin column, and (7) incubation of the 6-FAM-labeled ssDNA pool resulting from each SELEX cycle with GST-*Li*GP63m beads, to start a new cycle. A counter-selection step, performed every three cycles, is indicated with purple boxes, where (a) the ssDNA purified from one cycle is incubated with GST-beads, (b) ssDNA sequences bound to GST beads are removed, and (c) the unbound sequences are incubated with GST-*Li*GP63m beads to start a new cycle. After seven cycles (plus three counter-selection steps), the aptamer pool was cloned and sequenced. (**C**) Flow cytometry analysis of the binding of 6-FAM-labeled aptamer pools from cycles 2, 4 and 7 to *L. infantum* promastigotes. The randomly selected unspecific Apt700 sequence [34] was included as negative control.

GP63 is encoded by eight genes in *L. infantum* [13], GP63-2 (LINF_100010200) being the isoform used here for aptamer selection. The mature form of *L. infantum* GP63 (*Li*GP63m) was used due to its high sequence homology with the crystallized mature GP63 from *L. major* [10] and with most GP63 isoforms encoded in *L. infantum*, supporting its use as a representative isoform for this proof-of-concept study. *Li*GP63m, spanning from Val97 to Asn574 and lacking the N-terminal propeptide that keeps the enzyme inactive during translation (Figure 2A), was cloned into the pGEX-5X-1 vector and expressed in C41 *E. coli* with a GST tag to facilitate protein purification (Figure S1A). GST-*Li*GP63m bound to Sepharose–glutathione beads was used as a target for a bead-based SELEX process (Figure 2B). Specific DNA aptamers targeting *Li*GP63m were selected from an initial ssDNA library after several SELEX rounds. The forward primer used for PCR amplification was labeled with 6-carboxyfluorescein (6-FAM), allowing for the binding of the aptamer pools to fixed *L. infantum* promastigotes to be tracked using flow cytometry (Figure 2C). As observed, the 6-FAM-ssDNA pool from cycle 7 targeted a significantly higher proportion of the promastigote population (65%) than the cycle 2 pool (5%) or the negative control aptamer Apt700 (5%), indicating a clear enrichment in oligonucleotide sequences binding *Li*GP63m in the target cells.

### 3.2. Isolation and Characterization of Individual LiGP63m-Specific Aptamers

After confirming its specificity for the target cells, the enriched oligonucleotide pool from cycle 7 was cloned into the pJET1.2/blunt plasmid and used to transform *E. coli* DH5α cells. Twenty colonies were isolated and their plasmid inserts sequenced (Table 1); the resulting aptamers will be henceforth named *Li*GP63Apt-N, where N corresponds to the respective original colonies. To confirm that the aptamers specifically recognized the target protein and not the GST tag, *Li*GP63m was cloned into the pCold^TM^ TF plasmid and expressed in C43 *E. coli* as a 6 × histidine-tagged protein fused to a trigger factor to increase solubility (henceforth referred to as His-TF-*Li*GP63m; Figure S1B). The purified protein was then used in dot blot assays probed with the 20 aptamer sequences synthesized carrying a 6-FAM group in their 5’ end (Figure S2A). Different degrees of aptamer specificity were observed according to densitometry analysis (Figure S2B), with *Li*GP63Apt-23, -27 and -28 being the sequences with the highest binding to the target protein. Afterwards, the aptamer binding affinities for fixed *L. infantum* promastigotes were estimated using fluorescence confocal microscopy (Figure S3) and flow cytometry (Figure S4). All individual aptamers showed a targeting to fixed *L. infantum* promastigotes, with binding percentages ranging from 60% to 94% based on the quantitative data obtained from the flow cytometry analysis. The aptamer ranking, from the highest to the lowest degree of targeting, was as follows: *Li*GP63Apt-27>-10>-4>-16>-7>-22>-1>-28>-30>-20>-19>-23>-29>-24>-15>-2>-3>-25>-9>-13. When the propensity to form G-quadruplexes, planar arrays of four guanines which result in stable secondary structures [53], was computationally predicted, 14 of the 20 selected aptamers had a strong likelihood to form them, with a G-score [54] ≥ 38 (Table 1).

**Figure 3:**
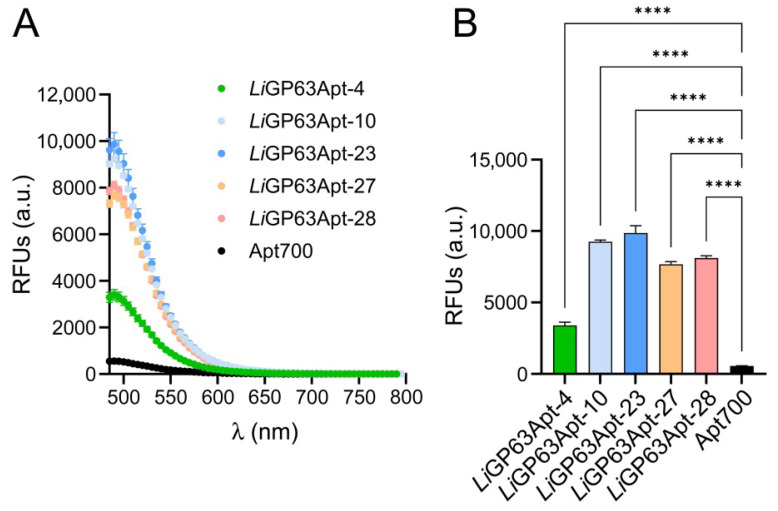
ThT assay for the detection of G-quadruplex structures. (**A**) Relative fluorescence emission intensity of anti-*Li*GP63 aptamers treated with ThT. Apt700 was included as a negative control of aptamer not forming G-quadruplexes. Aptamers and ThT were used at a concentration of 1 µM. (**B**) Statistical significance analysis of RFU values at the 490 nm peak compared to the negative control aptamer, calculated using one-way ANOVA followed by Dunnett’s test. **** *p* < 0.0001; RFUs: relative fluorescence units; a.u.: arbitrary units.

Among the 20 aptamers from Table 1, those three with the highest binding affinity to the purified His-TF-*Li*GP63m according to dot blot assays (*Li*GP63Apt-23, -27 and -28) and those binding to >90% *L.infantum* promastigotes according to flow cytometry (*Li*GP63Apt-4, -10 and -27) were selected for further analysis, *Li*GP63Apt-27 being the only one meeting both criteria. Computational analysis [55] indicated that these five selected sequences displayed features characteristic of functional aptamers, such as secondary structures containing single-stranded regions, hairpins, and duplex stems (Supplementary Table S1). When the potential presence of G-quadruplexes was empirically evaluated using a ThT-based assay, the fluorescence signal for all five selected aptamers was 3- to 10-fold higher than for the Apt700 negative control (Figure 3), supporting the validity of the in silico predictions.

**Figure 4:**
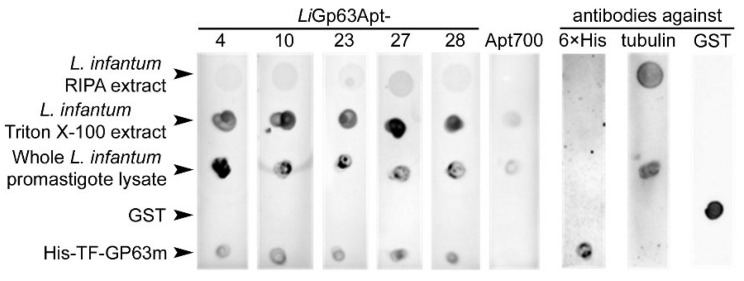
Dot blot assay to determine the specificity of the selected aptamers towards *Li*GP63. His-TF-*Li*GP63m, GST, whole *L. infantum* promastigote lysate and Triton X-100 and RIPA extracts were dotted on a nitrocellulose membrane (1.25 µg of protein in 2.5 µL) and incubated with 600 nM of 6-FAM-labeled aptamers or with antibodies, whose binding was detected as indicated in the Section 2. Apt700 was used as an unspecific aptamer control. Anti-tubulin confirmed the presence of cytoskeleton components in *L. infantum* total lysate and in RIPA extract.

In dot blot assays (Figure 4), all five aptamers detected *Li*GP63 in whole *L. infantum* promastigote lysates and in Triton X-100 extracts, which contained the membrane-associated proteins, in agreement with the expected location of *Li*GP63 in the plasma membrane. Accordingly, *Li*GP63 was not detected by any aptamer in RIPA extracts containing cytoskeletal and nuclear proteins. The negative control aptamer Apt700 gave only a weak signal in the whole cell lysate and the Triton X-100 extract, possibly reflecting unspecific interactions.

**Figure 5:**
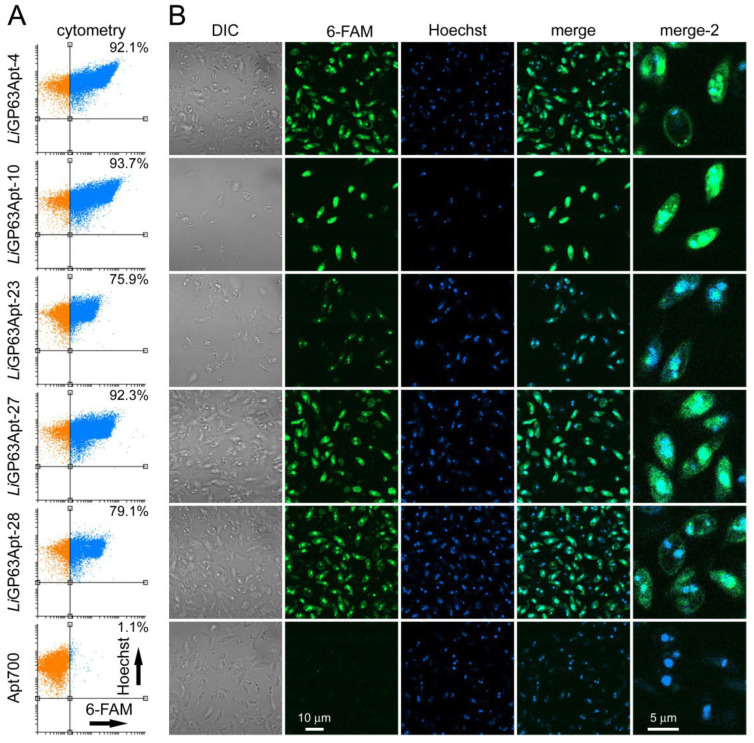
Targeting analysis of the five 6-FAM-labeled selected aptamers to fixed *L. infantum* promastigotes, studied by (**A**) flow cytometry and (**B**) confocal fluorescence microscopy. Cytometry panels show arbitrary fluorescence units. Percentages in (**A**) indicate the fraction of promastigotes positive for 6-FAM (upper right quadrant in each panel, blue color). DIC: differential interference contrast. Merge refers to Hoechst 33342 and 6-FAM channels only; merge-2 panels are zoomed regions of the corresponding merge panels. Scale bar is 10 µm for all microscopy panels, except for those in the last column, where it is 5 µm.

Flow cytometry targeting analysis of the five selected aptamers showed specific binding to *L. infantum* promastigotes (>75%, Figure 5A), which correlated well with the fluorescence microscopy observations (Figure 5B). The Apt700 negative control exhibited minimal targeting according to flow cytometry (1.1%) and did not display any detectable fluorescence signal, whereas the *Li*GP63-specific aptamers produced a strong 6-FAM fluorescence. Remarkably, exposure to different aptamers resulted in different subcellular fluorescence patterns, ranging from a diffuse cytoplasmic distribution (*Li*GP63Apt-10 and -27) to a pronounced membrane association (*Li*GP63Apt-4, -27 and -28) or localization to small perinuclear regions (*Li*GP63Apt-23). These differences probably reflected the binding of individual aptamer sequences to distinct regions of *Li*GP63, which might be differently exposed within the cell. Although the majority of GP63 is located at the parasite surface (ca. 75%), a small fraction (ca. 1.5%) remains in the endoplasmic reticulum, and the rest is cleaved from the membrane or released to the extracellular space via vesicles [13]. It is likely that this export transit to the cell membrane is responsible for the cytoplasmic signal observed with some aptamers. Because aptamers are single-stranded DNA molecules, their potential association with DNA-containing compartments, including the nucleus and kinetoplast, was assessed. Colocalization analysis using Manders’ coefficient (Supplementary Table S2) revealed values ranging from 0.29 to 0.54, indicating that a portion of the aptamer signal overlaps with Hoechst 33342 staining. These results suggest that, upon fixation, some aptamer molecules interact with nuclear and kinetoplast DNA, while a substantial fraction remains outside these compartments.

### 3.3. Aptamer Binding Affinity Characterization

The apparent *B_max_* and *K_D_* and the binding affinity for *Li*GP63m of the selected aptamers was assessed through the incubation of different concentrations of the 6-FAM-labeled aptamers with a fixed amount of His-TF-*Li*GP63m (2.5 µg) bound to latex beads (Figure S5 and Table 2). The aptamer with the highest *B_max_* was *Li*GP63Apt-28, indicating that it was able to bind more sites within the protein than the other aptamers, whereas the lowest value for the apparent *K_D_* (i.e., highest affinity) corresponded to *Li*GP63Apt-27, although all five aptamers had apparent *K_D_* values in the one-digit µM range. The aptamer with the highest apparent binding affinity (*B_max_*/*K_D_*) was also *Li*GP63Apt-27.

**Figure 6:**
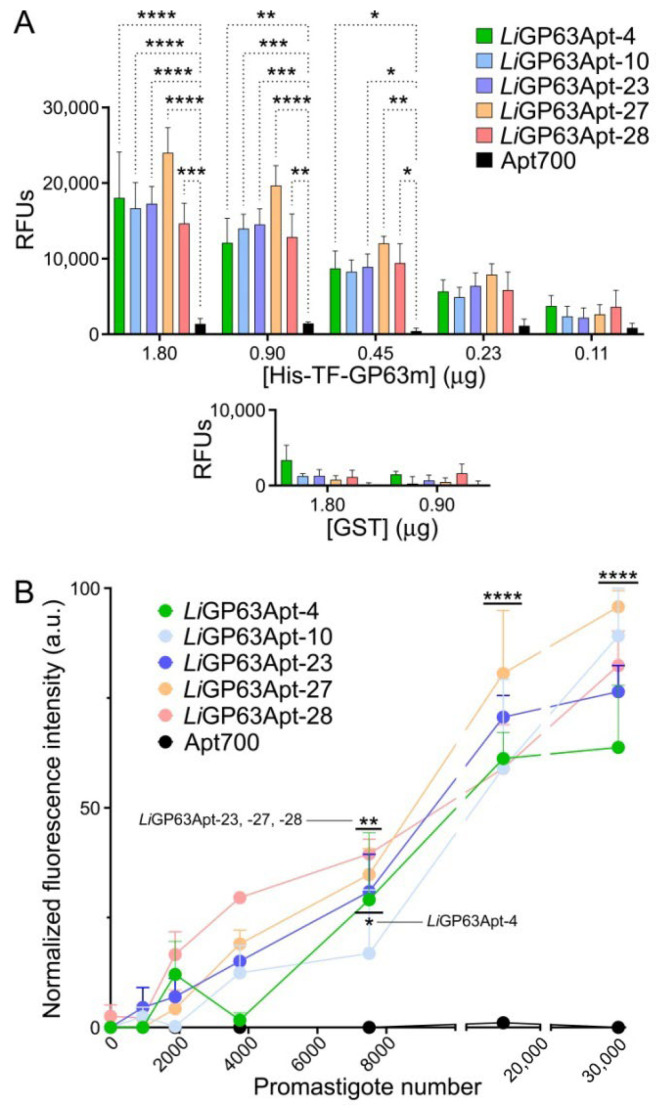
ALISA analysis of the limit of detection of the selected aptamers for *Li*GP63m and *L. infantum* promastigotes. (**A**) Sensitivity of the aptamers for the detection of His-TF-*Li*GP63m. The immobilized protein amount varied between 1.8 and 0.11 µg, while the concentration of aptamer was fixed at 600 nM. GST was incubated at the two highest protein concentrations used (1.8 and 0.9 µg) as a negative binding control. RFUs: relative fluorescence units. Average ± SEM of three different experiments is shown (*n* = 3). (**B**) Sensitivity of the selected aptamers for the detection of *L. infantum* promastigotes. Assays were done with total lysate from *L. infantum* promastigotes that corresponded to a number of parasites ranging from 938 to 30,000. Average ± SEM of three different experiments is shown (*n* = 3). Statistical significance compared to the unspecific Apt700 was calculated using one-way ANOVA followed by Dunnett’s test (* *p* < 0.05; ** *p* < 0.01; *** *p* < 0.001; **** *p* < 0.0001).

The limit of detection of the aptamers for their target protein was determined in an aptamer-linked immobilized sorbent assay (ALISA, [51]) through the incubation of a fixed concentration of the selected 6-FAM-labeled aptamers (600 nM) with serial dilutions of His-TF-*Li*GP63m (from 1.8 to 0.11 µg). *Li*GP63Apt-4, -23, -27 and -28 were able to detect down to 0.45 µg of protein (Figure 6A). As a proof-of-concept to explore the potential use of the selected aptamers as diagnostic tools, an ALISA was performed with a whole parasite lysate, where the amount of protein was used to determine the corresponding number of promastigotes [42]. Aptamers *Li*GP63Apt-4, -23, -27 and -28 were capable of specifically detecting down to 7500 promastigotes (Figure 6B). In the assayed conditions, the limit of detection of *Li*GP63Apt-10 was 15,000 promastigotes. Although not statistically significant, at 2000 and 4000 promastigotes, we observed a clear difference in the normalized fluorescence intensity between the control aptamer and *Li*GP63Apt-28, -27, and -23, with *Li*GP63Apt-28 consistently showing the highest signal (Figure 6B). This result aligns with its highest apparent $B_{max}$ value from the saturation binding analysis (Table 2), suggesting a higher number of binding sites, which enables enhanced antigen capture. However, according to the binding equation B = *B_max_* · L/(*K_D_* + L), where B is the bound ligand concentration and L the free ligand concentration, the superior binding potential of *Li*GP63Apt-27 (*B_max_*/*K_D_* ratio) predicts a better ligand recognition at lower parasitemias. Nonetheless, to solve this apparent discrepancy and confirm the relative sensitivities, more precise methods, such as surface plasmon resonance, will be needed to confirm the real values of *K_D_* and *B_max_*, along with more sensitive methods to determine the limit of detection of the parasites.

### 3.4. Effect of Aptamers in the Infection of Macrophages by L. infantum

**Figure 7:**
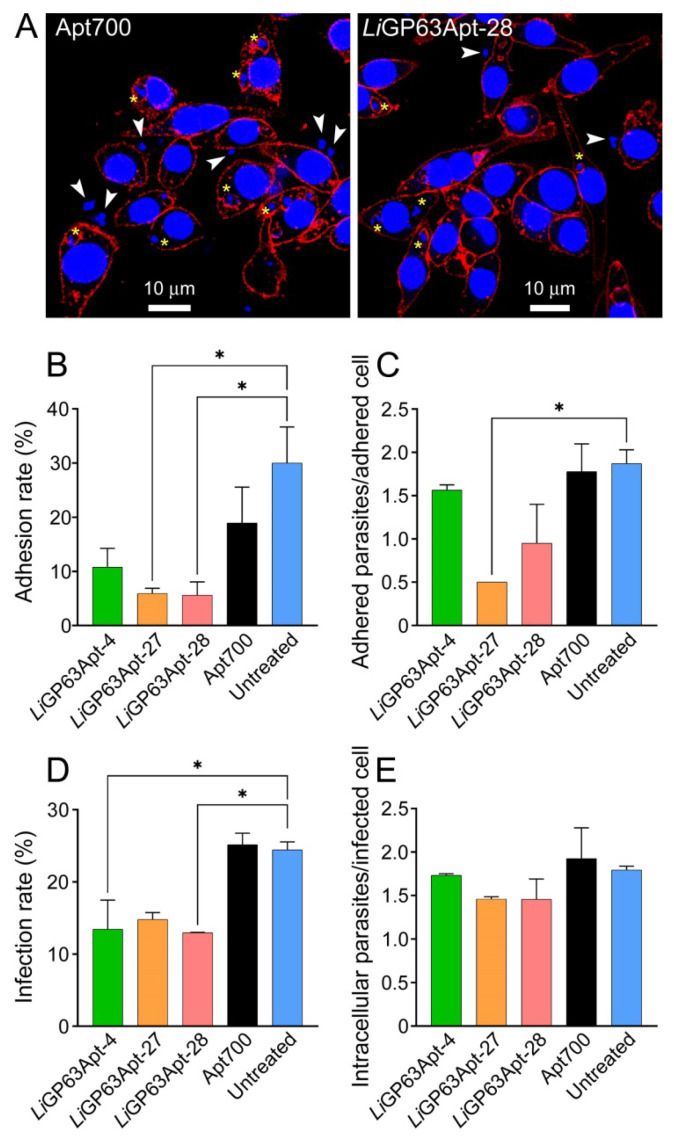
Confocal fluorescence microscopy analysis of the effect of anti-*Li*GP63 aptamers on *L. infantum* adhesion to and intake by macrophages. Promastigotes were preincubated for 1 h with 1 µM of the indicated aptamers prior to infection of RAW 264.7 macrophages. Cells were incubated overnight, fixed, and imaged through confocal fluorescence microscopy, and finally parasite adhesion to and internalization in macrophages was quantified. (**A**) Representative confocal fluorescence microscopy images of the samples treated with Apt700 and with *Li*GP63Apt-28, showing *L. infantum* parasites adhered to or internalized within macrophages. White arrowheads indicate parasites in contact with macrophage membranes, and yellow asterisks indicate *L. infantum* inside macrophages. (**B**) Adhesion rate, expressed as the percentage of macrophages carrying at least one adhered parasite. (**C**) Adhered parasites per adhered cell (average number of parasites attached to macrophages with at least one adhered parasite). (**D**) Infection rate, expressed as the percentage of macrophages carrying at least one intracellular *L. infantum* amastigote. (**E**) Intracellular parasites per infected cell (average parasite number per infected macrophage). For each condition, 100 cells were analyzed. Mean values ± SEM of two different experiments are shown (*n* = 2). Statistical significance compared to macrophages exposed to untreated promastigotes was calculated using one-way ANOVA followed by Dunnett’s test. * *p* < 0.05.

The aptamers *Li*GP63Apt-4, -27 and -28 were used to evaluate, through confocal fluorescence microscopy, their potential for reducing the parasite adhesion to and infection of macrophages (Figure 7). When RAW 264.7 macrophages were exposed to *L. infantum* promastigotes pre-incubated for 1 h with 1 µM of pre-folded *Li*GP63Apt-4, -27 or -28 aptamers, a significant reduction was observed for *Li*GP63Apt-27 and -28 in the adhesion rate (fraction of macrophages in close contact with at least one parasite) compared to control cells exposed to *L. infantum* promastigotes non-treated or pre-treated with the Apt700 unspecific aptamer (Figure 7B). In contrast, the average number of adhered parasites per adhered macrophage was significantly reduced only for *Li*GP63Apt-27 (Figure 7C). Similarly, a clear reduction in the infection rate (fraction of macrophages with at least one intracellular parasite) was detected when parasites were pre-treated with aptamers (Figure 7D). However, the average number of internalized parasites per infected macrophage remained unchanged in aptamer-treated samples (Figure 7E), suggesting that, once parasites are internalized, their capacity to survive or replicate within macrophages is not significantly affected, and that the observed differences reflect interferences with parasite intake rather than with intracellular growth. An amount of 5 µM of either aptamer had no effect on the viability of promastigotes for up to 72 h of incubation (Figure S6), indicating that the observed decrease in infectivity was not due to promastigote death.

### 3.5. Assessment of Aptamers as Targeting Moieties of Liposomes

Aptamers *Li*GP63Apt-4 and -28, which significantly reduced macrophage infection rates by *L. infantum* promastigotes and bound *L. infantum* promastigote plasma membranes according to fluorescence microscopy images (Figure 5), were selected to test their potential as targeting elements on liposomes for the improved delivery of encapsulated drugs to promastigotes. Aptamers functionalized at their 5’ end with a thiol group were covalently conjugated onto maleimide-containing liposomes. Aptamer binding was assessed through a measurement of the change in the zeta potential of liposomes to a more negative value, and through the corresponding detection of DNA bound to them [56] (Table 3). Physicochemical characteristics like size and polydispersity index were maintained, indicating that the liposome stability was not affected by the conjugation of aptamers.

**Figure 8:**
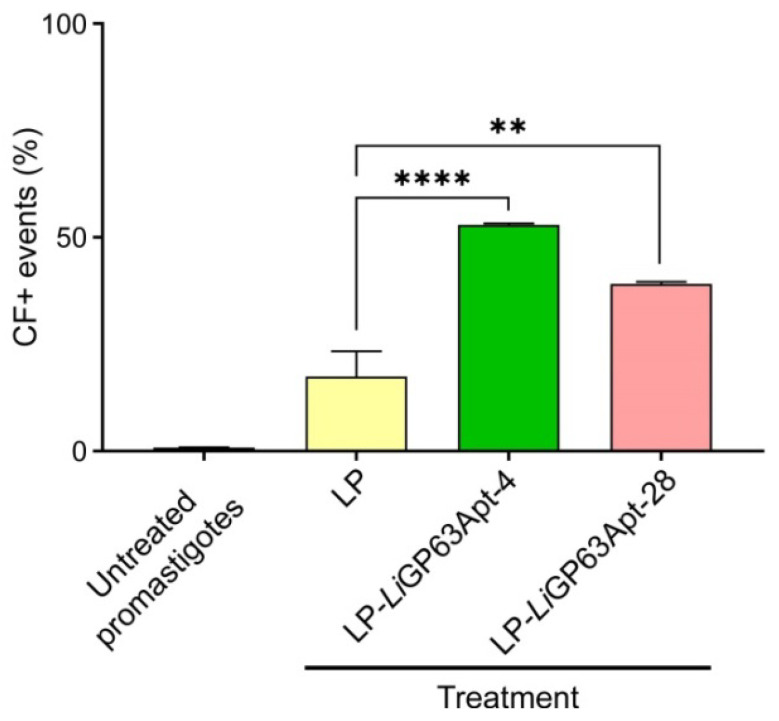
Liposome targeting to *L. infantum* promastigotes. Flow cytometry analysis of CF-PE-labeled liposomes, either plain or conjugated to *Li*GP63Apt-4 and -28, incubated with fixed *L. infantum* promastigotes. Mean values ± SD are indicated (*n* = 3). Statistical significance compared to untreated parasites was calculated using one-way ANOVA followed by Dunnett’s test. ** *p* < 0.01; **** *p* < 0.0001. CF: carboxyfluorescein; LP: liposome.

A flow cytometry assay was conducted to determine whether aptamers conjugated to liposomes could serve as targeting elements for nanocarrier-based drug delivery to *L. infantum* promastigotes. The targeting efficiencies of liposomes functionalized with *Li*GP63Apt-4 and *Li*GP63Apt-28 (LP-*Li*GP63Apt-4 and -28) were compared with those of plain liposomes (LP) after 3 h of incubation with fixed promastigotes. Liposome binding to promastigotes was detected by measuring the fluorescence of the lipid carboxyfluorescein–phosphatidylethanolamine (CF-PE), which was incorporated in the liposome formulation. The fluorescence signal was significantly higher when promastigotes were incubated with LP-*Li*GP63Apt-4 (*p* < 0.0001) or LP-*Li*GP63Apt-28 (*p* < 0.01), relative to those incubated with plain liposomes (Figure 8).

## 4. Discussion

Aptamers represent an attractive tool for biomedical research in diagnostic and therapeutic applications [57,58]. Within the leishmaniasis elimination framework, aptamers can be employed as ligands to functionalize nanocarriers for targeted drug delivery, as diagnostic tools for detecting specific antigens, to study molecular interactions between parasite and host cells, or as therapeutic agents operating through different mechanisms, such as inhibiting essential protein activities or blocking interactions between molecules. In this work, the five aptamers with highest binding affinity to the purified recombinant *Li*GP63protein in dot blots or best binding to *L. infantum* promastigotes in flow cytometry assays were among those with the highest G-scores. The formation of G-quadruplex structures in these aptamers was experimentally confirmed using a ThT-based fluorescence assay. Aptamers containing G-quadruplexes are particularly adequate for therapeutic, diagnostic and drug delivery applications [59], in part due to their thermodynamic and chemical stability, low immunogenicity and resistance to serum nucleases. Remarkably, G-quadruplexes are characterized by an enhanced cellular uptake [60], which is an important property regarding potential intracellular targets. *Li*GP63Apt-27, the aptamer with the highest G-score, was also the one with the best binding potential (i.e., with better ligand recognition at lower parasitemias).

Following the inoculation of *Leishmania* promastigotes in the dermis, parasite movement is facilitated by the extracellular matrix degradation mediated by GP63 [61]. Because the interaction of GP63 with macrophage receptors is crucial for *Leishmania* invasion [12], aptamers binding to GP63 may interfere with it, reducing parasite adhesion and subsequent internalization and thereby decreasing infection rates. This scenario aligns well with the results obtained in the present work (Figure 7), suggesting that aptamers may modulate the association between promastigotes and macrophages. Further validation of the aptamers in human macrophage cell lines should be addressed before eventual entry in the preclinical development pipeline.

An important element of the host innate response to infection by pathogens is complement-mediated lysis. The complement subunit C3b participates in the phagocytosis of *Leishmania* by macrophages through its interaction with the complement receptors CR3 or CR1 and fibronectin receptors [12,62,63,64]. The role of GP63 in the inactivation of the complement by increasing the conversion of C3b into the inactive complement fragment iC3b [65] is a key process that allows *Leishmania* survival in the mammalian host, as illustrated by the susceptibility to the complement-mediated lysis of *L. major* promastigotes deficient in GP63 [66]. In addition, complement inactivation has been shown to facilitate *L. major* capture by macrophages through increased phagocytosis by CR1, the C3b receptor [67]. Finally, the mammalian β-galactosidase-binding galectin-3, which has been described to be part of the antileishmanial host defense [68,69] through the binding of promastigotes [70], is cleaved by GP63, which prevents the formation of galectin-3 lattices that play a role in the initial immune response to *Leishmania* infection [71]. The aptamer interaction with GP63 in promastigotes might neutralize its activity, eventually increasing the susceptibility of *Leishmania* to complement, reducing macrophage entry, and increasing exposure to the immune system.

Although no GP63 inhibitors have reached preclinical studies, some of them have shown significant activity, such as Glycoside-2 (IC_50_ of 1.13 µM in *L. donovani* promastigotes [72]), benzimidazole compounds (IC_50_ of 0.62 to 0.92 µg/mL in *L. major* promastigotes [73]), and the 7-hydroxycoumarin derivative L1 compound (IC_50_ of 1.24 µM in *Leishmania amazonensis* promastigotes [74]). Given the scarcity of therapeutic candidates, GP63-binding aptamers that could eventually interfere with its multiple roles should be carefully evaluated as potential antagonists of this protein. Improved anti-GP63 therapeutic aptamers could be obtained with other SELEX designs screening for enzymatic activity inhibition instead of the protein binding-based assay used here. For this alternative strategy, the protease nature of GP63 should be harnessed to incorporate in each cycle a rapid method to select those aptamers inhibiting proteolytic activity.

The current diagnosis strategies for leishmaniasis consist either in the direct microscopic detection of the parasite or in molecular assays, which require specialized personnel and/or expensive equipment not available in most endemic regions. Serological tests are also useful, but only as a complementary method, since they cannot distinguish between current or past infections [2]. By exploiting the proteolytic activity of GP63 for diagnostic approaches, casein was conjugated to gold nanoparticles, which, upon interaction with GP63, enabled the detection of 0.55 parasites/mL [75]. However, because casein can interact with multiple molecules, developing specific aptamers against GP63 could provide a basis for the improved diagnosis of leishmaniasis. In this context, an aptamer-based sensor (aptasensor) stands out as one of the most promising options for transitioning from the bench to a commercially viable point-of-care testing device [37], as illustrated by the aptasensor designed for detecting *Plasmodium* lactate dehydrogenase [76].

Four of the aptamers developed in this work (*Li*GP63Apt-4, -23, -27 and -28) were capable of detecting endogenous *Li*GP63 in whole parasite lysates containing down to 7500 promastigotes. Although not statistically significant, *Li*GP63Apt-23, -27 and -28 showed markedly higher normalized fluorescence signals than the control aptamer down to 2000 promastigotes, suggesting a potential sensitivity beyond the reported limit of detection. This opens the door for exploring other diagnostic tools or optimized ALISA conditions (e.g., enzymatic amplification) to determine if this difference achieves statistical significance, as the direct fluorescence readout from aptamer probes, while sensitive, has a limited precision at the limit of detection. Indeed, when comparing the results of this study with previous works, such as those by the group of Víctor M. González, the limits of detection of 7500 parasites for *Li*H2A [42] and 6000 for *Li*H3 [43] were achieved using an enzyme-linked oligonucleotide assay (ELONA) approach. It is anticipated that an ELONA format could enhance detection capabilities due to the signal amplification provided by the enzyme. The eventual development of future aptamer-based diagnostic devices must consider the calculation of affinity constants in the biological fluids where target cells are to be detected. Aptamers with improved sensitivity for the detection of *Leishmania* could be obtained through whole-cell SELEX, a strategy that allowed the generation of aptamers against *Plasmodium falciparum*-infected red blood cells [34], whose affinities for intact cells were in the same order of magnitude as those determined here against recombinant purified *Li*GP63. The elucidation of the 3D structures of the aptamers, e.g., through AlphaFold 3 [77], could help to identify their binding sites on the surface of the protein using modeling or crystallization studies of the aptamer-GP63 complex, allowing for improved binding affinities through a chemical adaptation of the oligonucleotide sequences.

The localization of *Leishmania* amastigotes within phagolysosomes compromises the access of antileishmanial drugs, requiring long and painful treatment regimens [78]. To overcome this problem, targeted delivery has been reported to ameliorate drug bioavailability, reduce cytotoxicity, expand release kinetics, and improve cellular uptake [79,80]. The functionalization of liposomes with aptamers has been proven to increase targeting to specific cells, boosting the efficacy of encapsulated drugs, such as cisplatin [81] or 5-fluorouracil [82]. The results reported here indicate that liposomes conjugated to anti-*Li*GP63 aptamers had an improved targeting to promastigotes, thereby potentially enhancing the efficacy of liposome-based drug delivery systems. The covalent link to lipids did not abolish targeting despite the presumed conformational restriction imposed by this type of crosslinking, which suggests that aptamers developed and characterized in solution will maintain most of their selective binding potential when incorporated in nanocarriers designed for targeted drug delivery. Drug leakage and intracellular concentration analyses will be required in the future to fully characterize the therapeutic potential of such nanocapsules.

## 5. Conclusions

The initial objective of this work was to explore if aptamers generated against an essential *Leishmania* protein that is abundantly expressed and extracellularly exposed on promastigotes could be used for diagnostic and therapeutic applications. The results presented above indicate that DNA aptamers can be selected with a high in vitro affinity and specificity for the GP63 protein of *Leishmania* parasites. These aptamers reduce the intake by macrophages of *L. infantum* promastigotes, detect the protein in live parasites, and can be used to functionalize liposomes for their improved binding of the pathogen. DNA aptamers hold promise in future leishmaniasis control strategies as potential diagnostic and therapeutic tools and as targeting molecules for drug delivery nanocarriers. It will be important to assess the binding characteristics of the aptamers to GP63 proteins from other *Leishmania* species to ensure that the resulting therapeutic and/or diagnostic product will be universally applicable.

## Figures and Tables

**Table 1:** Sequences and G-scores of the 20 aptamers selected from the pool obtained after 7 SELEX cycles. The sequence of the unspecific aptamer Apt700 [34] is included (shadowed in gray). For those sequences where more than one G-score was obtained, only the highest is indicated.

Aptamer	Aptamer Sequence	G-Score
*Li*GP63Apt-1	ATACCAGCTTATTCAATTATCGGGGGTGGCCAGGTGGGAGGGTGGGCGGGTGCTGGTAAGATAGTAAGTGCAATCT	42
*Li*GP63Apt-2	TTACCAGCTTATTCAATTGCACATAGGGGGATGGGTGGGTGGGGCTATGTGGTTAGGCAGATAGTAAGTGCAATCT	41
*Li*GP63Apt-3	ATACCAGCTTATTCAATTACGACCTGACCCAACATCCCGTGCCCCCGTTTAGTAACCGAGATAGTAAGTGCAATCT	-
*Li*GP63Apt-4	TTACCAGCTTATTCAATTCGTCGGTGGGTGGGTCGGGTGGGGACGAAGTGACGTTTCAAGATAGTAAGTGCAATAT	41
*Li*GP63Apt-7	ATACCAGCTTATTCAATTACGGGGACCGTGGGTGGGTGGGGGGGAGGTCCATGGCTTAAGATAGTAAGTGCAATCT	42
*Li*GP63Apt-9	ATACCAGCTTATTCAATTGCCAGCATATAATAACCGGGCCATACCGCTAACGTCTACCAGATAGTAAGTGCAATCT	-
*Li*GP63Apt-10	TTACCAGCTTATTCAATTCCATCGGGCGGGGGGGTGGGTAGCATGGAATCAGAGTCGTAGATAGTAAGTGCAATAT	42
*Li*GP63Apt-13	ATACCAGCTTATTCAATTATCACAGGCAACACACGCCCGCGGGCTCGCGACGGTAGTGTTGAGATAGTAAGTGCAATCT	-
*Li*GP63Apt-15	ATACCAGCTTATTCAATTGGGGCGGGCGGGATACGACCTTATTTACCTGCTACTCAGATAGTAAGTGCAATC	19
*Li*GP63Apt-16	ATACCAGCTTATTCAATTCGGGATGGGTGGGCGGGGGTGGCGGTTCGGTATGGGGCTTAGATAGTAAGTGCAAATC	41
*Li*GP63Apt-19	ATACCAGCTTATTCAATTCGTGCGGGAGGGTGGTACGGGGTGGGAGGCGGGCTGCTGGAGATAGTAAGTGCAATCT	38
*Li*GP63Apt-20	ATACCAGCTTATTCAATTACGGGCGGGGGCGGGCGGGTGGGGTGCGGCAATTCTCTGGAGATAGTAAGTGCAATCT	42
*Li*GP63Apt-22	ATACCAGCTTATTCAATTACCGAGGGTGGGGCGGGGGGGCGGGGGAGGAGGAGGTCAGTGCCTCAAGATAGTAAGTGCAATC	42
*Li*GP63Apt-23	ATACCAGCTTATTCAATTCTGGTGGGGGGGGCAGGGCGGGTCATACTGCATTACTTGGAGATAGTAAGTGCAATCT	41
*Li*GP63Apt-24	ATACCAGCTTATTCAATTACCGGGGGGGGAGGGTGGGTCCTGGCGGAAAATGGCGCGGAGATAGTAAGTGCAATCT	17
*Li*GP63Apt-25	ATACCAGCTTATTCAATTGCTAGGTCACGCACTGGGGTGGGTTGGGTGGGAGTTTACCAGATAGTAAGTGCAATCT	-
*Li*GP63Apt-27	TTACCAGCTTATTCAATTGCGGGGGGGGGGGGGGGGTGGAGGGGGTTGTAACTGTGAGAGATAGTAAGTGCAATCT	80
*Li*GP63Apt-28	ATACCAGCTTATTCAATTGTACGCGTGTGGGGGTGGGGGGGCGGGTTCATACGGTGAAGATAGTAAGTGCAATCT	42
*Li*GP63Apt-29	TTACCAGCTTATTCAATTGACGAGAATCGGGGCGGGCGGGGGGGGTAAGCATGATCGGAGATAGTAAGTGCAATCT	42
*Li*GP63Apt-30	ATACCAGCTTATTCAATTGTGCAGGGGTGTGGGTGGGTGGGCGGGTACGGCTAAGGGTAGATAGTAAGTGCAATCT	42
Apt700	ATACCAGCTTATTCAATTAGTTGTGGTTGCAACTTTTTATTATTTGTTCGTATCTTTAAGATAGTAAGTGCAATCT	-

**Table 2:** Apparent *K_D_*, *B_max_* and binding potential (*B_max_*/*K_D_*) for the selected aptamers, determined from the data obtained in the experiment reported in Figure S5.

Aptamer	Apparent *K_D_* (µM)	Apparent *B_max_* (a.u.)	*B_max_*/*K_D_*
*Li*GP63Apt-4	0.6	3187	4903
*Li*GP63Apt-10	0.6	8753	14,836
*Li*GP63Apt-23	0.7	14,097	20,430
*Li*GP63Apt-27	0.3	8399	31,107
*Li*GP63Apt-28	2.1	24,242	11,544
Apt700 ^1^	−0.03	434	−14,467

^1^ The apparent *K_D_* value for Apt700 had a negative value because of the incorrect fitting in the binding curve data (Figure S5) due to the low affinity for His-TF-*Li*GP63m of this control aptamer. a.u.: arbitrary units.

**Table 3:** Determination of size, polydispersity index (PDI), zeta potential and DNA concentration of liposome suspensions, containing 5 mM lipid, with and without aptamer functionalization. The values are expressed as the mean ± SD (*n* = 3). LP: plain liposomes.

	Size (nm) ± SD	PDI ± SD	Zeta Potential (mV) ± SD	[ssDNA] (ng/µL) ± SD
LP	154.7 ± 4.1	0.12 ± 0.01	−7.55 ± 0.26	0.00 ± 0.00
LP-*Li*GP63Apt-4	141.1 ± 1.2	0.13 ± 0.03	−9.90 ± 0.98	41.50 ± 6.50
LP-*Li*GP63Apt-28	114.0 ± 0.6	0.13 ± 0.03	−9.14 ± 1.97	68.50 ± 6.61

## Data Availability

All the data supporting the reported results can be found in the main article and in the Supplementary Materials. Further data are available on request from the corresponding authors.

## Supplementary Materials

The following are available online at https://www.mdpi.com/article/10.3390/pharmaceutics18030304/s1, Figure S1: Expression and purification of *Li*GP63m and Western blot validation. Scheme of (A) pGEX-5X-*Li*GP63m and (B) pColdTF-*Li*GP63m plasmids containing the *Li*GP63m gene. Figure S2: Dot blot analysis of the binding affinity of the individual aptamer sequences for His-TF-*Li*Gp63m. (A) Dot blot. Five µL containing 2.4 µg of His-TF-*Li*Gp63m were dotted on a nitrocellulose membrane, subsequently blotted overnight with 600 nM of each 6-FAM labelled aptamer or with a dilution (1:2500) of an anti-His tag antibody. (B) Quantitative analysis with ImageJ [83] of the dot blot results from panel A, by normalizing the intensity of each dot to that of the anti-His tag antibody (black bar), to which it was assigned an arbitrary value of 100%. The orange bars indicate the aptamers chosen for further characterization. Figure S3: *L. infantum* promastigotes fixed and stained with 1 µM of 6-FAM-labeled aptamers. Figure S4: Flow cytometry analysis to quantify aptamer targeting to *L. infantum* fixed promastigotes during an overnight incubation with 1 µM of 6-FAM-labelled aptamers. Figure S5: Aptamer binding affinity characterization. Figure S6: Aptamer *in vitro* growth inhibition assay in *L. infantum* promastigote culture. Table S1: Prediction of secondary structures (at 22 °C and 137 mM Na^+^) in the five selected aptamers according to RNAfold (ViennaRNA package; rna.tbi [55]). Table S2: Manders’ coefficients of the colocalization of fluorescein and Hoechst 33342 signals in the fluorescence microscopy images from Figure 5B.
