# Supplementary material for: Development of DNA Aptamers Against Leishmania infantum GP63 Protein for Therapeutic and Diagnostic Applications
Source: Pharmaceutics. 2026 Feb 28;18(3):304. doi: 10.3390/pharmaceutics18030304 (PMC13029188; doi:10.3390/pharmaceutics18030304)
Supplement: Supplementary file 1 [file pharmaceutics-18-00304-s001.zip › pharmaceutics-4091315-supplementary.pdf]

Supplementary Material

Development of DNA Aptamers Against *Leishmania infantum* GP63 Protein for Therapeutic and Diagnostic Applications

Lucía Román-Álamo, Daniela Currea-Ayala, Gabriel S. Oliveira, Antonino Nicolò Fallica, Timen Mooren, Yunuen Avalos-Padilla and Xavier Fernàndez-Busquets

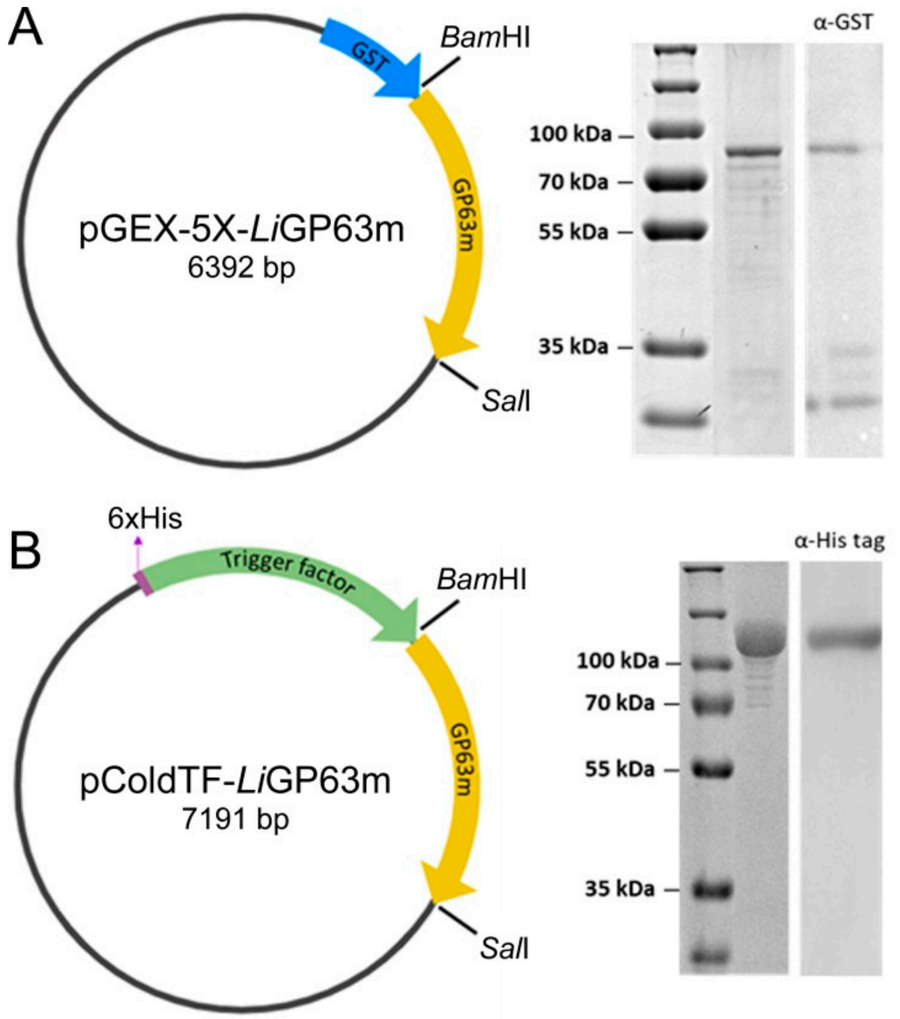

Figure S1. Expression and purification of *Li*GP63m and Western blot validation. Scheme of (A) pGEX-5X-*Li*GP63m and (B) pColdTF-*Li*GP63m plasmids containing the *LiGP63m* gene.

Right panels: SDS-PAGE gels stained with Coomassie blue showing the fractions purified in an ÄKTA pure™ chromatography system for (A) GST-*Li*GP63m bound to Sepharose glutathione-beads after its induction in C41 *E. coli* transformed with pGEX-5X-*Li*GP63m and (B) His-TF-*Li*GP63m after its expression in C43 *E. coli* transformed with the pColdTF-*Li*GP63m construct. The third lanes in each panel show Western blot assays using the antibodies *α*-GST and *α*-His tag. The expected molecular weights were 77 kDa for GST-*Li*GP63m (51 kDa from GP63m and 26 kDa from the GST tag) and 103 kDa for TF-*Li*Gp63m (51 kDa from GP63m and 52 kDa from the His-TF tag).

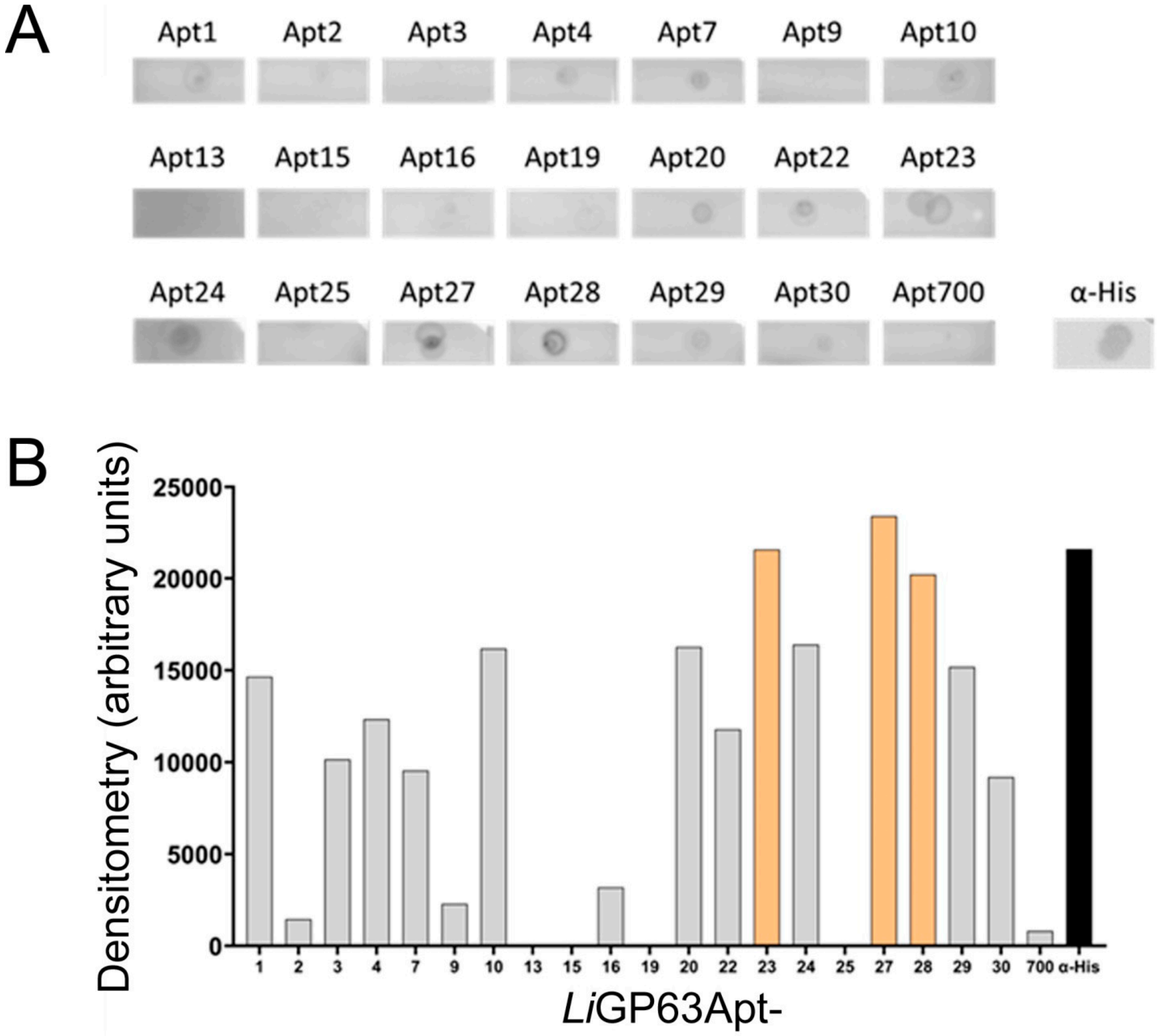

Figure S2. Dot blot analysis of the binding affinity of the individual aptamer sequences for His-TF-*Li*Gp63m. (A) Dot blot. Five μL containing 2.4 μg of His-TF-*Li*Gp63m were dotted on a nitrocellulose membrane, subsequently blotted overnight with 600 nM of each 6-FAM labelled aptamer or with a dilution (1:2500) of an anti-His tag antibody. (B) Quantitative analysis with ImageJ [83] of the dot blot results from panel A, by normalizing the intensity of each dot to that of the anti-His tag antibody (black bar), to which it was assigned an arbitrary value of 100%. The orange bars indicate the aptamers chosen for further characterization.

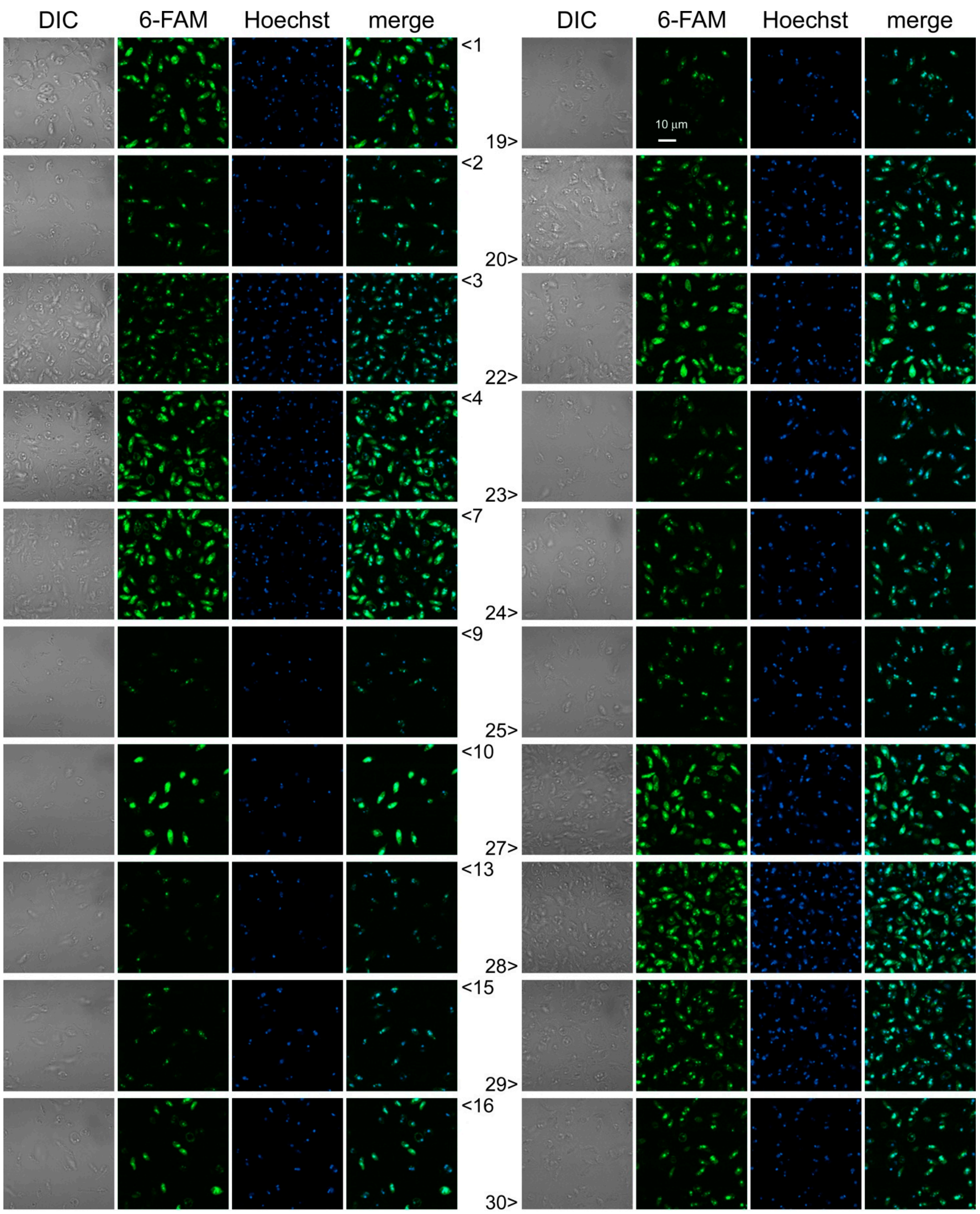

Figure S3. *L. infantum* promastigotes fixed and stained with 1 µM of 6-FAM-labelled aptamers. Promastigote nuclei were stained with Hoechst 33342 (blue). Merge refers to 6-FAM-aptamers and nuclei signals. Scale bar: 10 µm. DIC: differential interference contrast image.

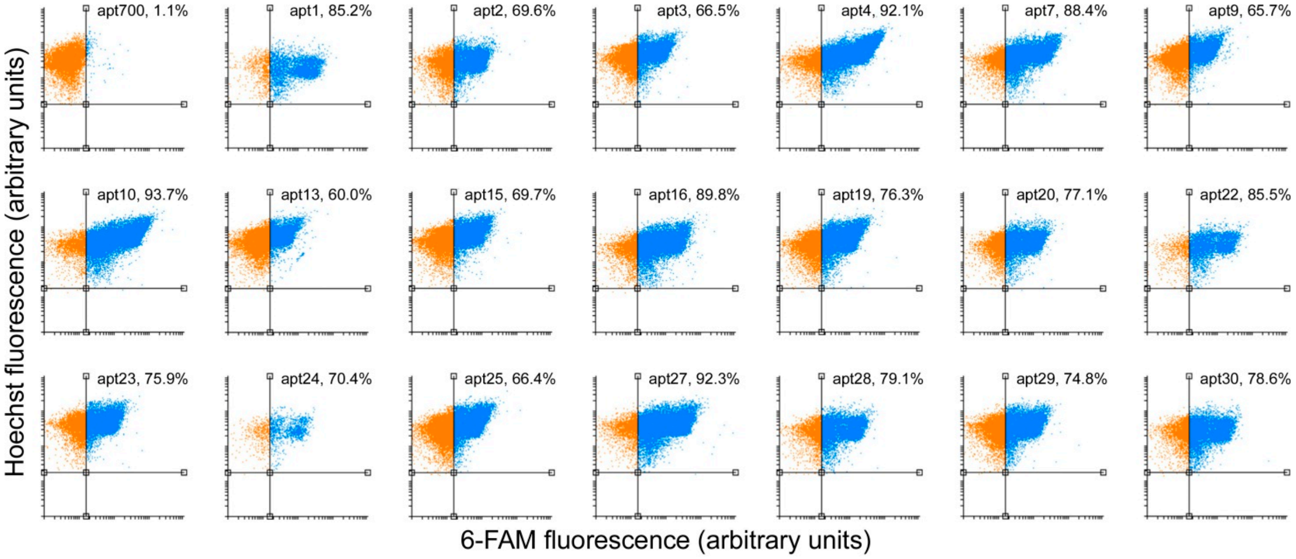

Figure S4. Flow cytometry analysis to quantify aptamer targeting to *L. infantum* fixed promastigotes during an overnight incubation with 1 µM of 6-FAM-labelled aptamers.
Percentages indicate for each aptamer the fraction of targeted promastigotes (i.e., those positive for 6-FAM fluorescence according to the set threshold). apt: abbreviation for "*Li*GP63Apt-" (except for apt700).

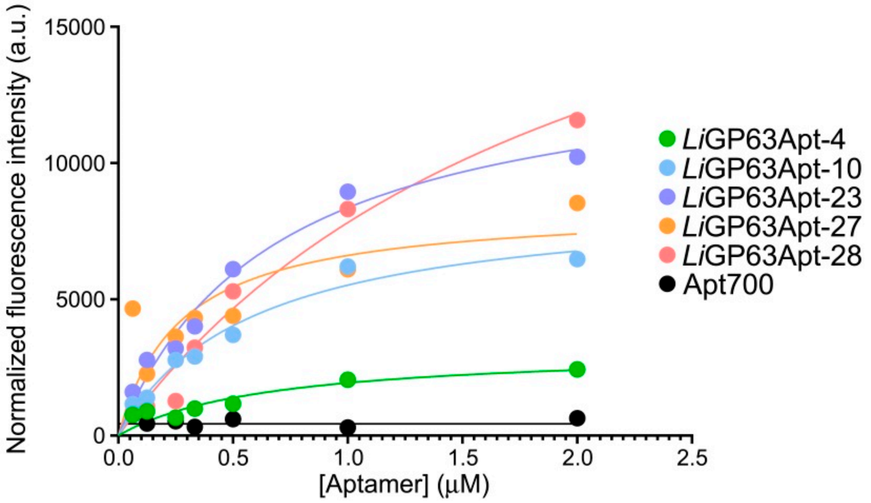

Figure S5. Aptamer binding affinity characterization.
The His-TF-*Li*Gp63m protein amount was fixed at 2.5 µg, while the concentration of the aptamer varied between 2.0 and 0.06 µM. a.u.: arbitrary units.

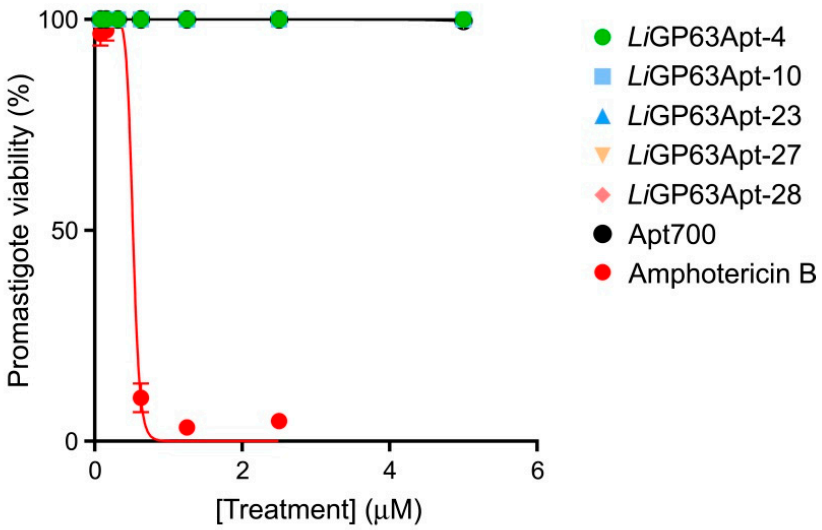

Figure S6. Aptamer *in vitro* growth inhibition assay in *L. infantum* promastigote culture.

Aptamers were incubated with the parasites for 72 h. All the aptamer curves are behind that of *Li*Gp63Apt4 (none of them affected promastigote viability up to 6 µM). Amphotericin B was used as a positive control. Data are presented as mean ± SD.

Table S1. Prediction of secondary structures (at 22 °C and 137 mM Na^+) in the five selected aptamers according to RNAfold (ViennaRNA package; rna.tbi [55]).
Numerical values (0–3) indicate the number of predicted structures of each type, where 0 corresponds to the lowest propensity to form a given structure and 3 to the highest.

Aptamer	ΔG (kcal/mol)	single-stranded	hairpin	internal loop	bulge	duplex	multi-branched
4	–6.69	2	1	0	0	1	0
10	–3.95	3	1	0	1	1	0
23	–8.93	3	3	0	0	3	0
27	–3.85	3	3	0	0	3	0
28	–4.37	3	2	0	0	2	0

Table S2. Manders' coefficients of the colocalization of fluorescein and Hoechst 33342 signals in the fluorescence microscopy images from Figure 5B.

**Aptamer**	**Manders' coefficient**
*Li*GP63Apt-4	0.53
*Li*GP63Apt-10	0.31
*Li*GP63Apt-23	0.29
*Li*GP63Apt-27	0.54
*Li*GP63Apt-28	0.32
Apt700	0.26
